# Supplementary material for: Nuclear FAK Controls Chemokine Transcription, Tregs, and Evasion of Anti-tumor Immunity
Source: Cell. 2015 Sep 24;163(1):160–73. doi: 10.1016/j.cell.2015.09.001 (PMC4597190; doi:10.1016/j.cell.2015.09.001)
Supplement: Document S1. Supplemental Experimental Procedures and Table S2 [file mmc1.pdf]

Cell

Supplemental Information

Nuclear FAK Controls Chemokine Transcription, Tregs, and Evasion of Anti-tumor Immunity

Alan Serrels, Tom Lund, Bryan Serrels, Adam Byron, Rhoanne C. McPherson, Alexander von Kriegsheim, Laura Gómez-Cuadrado, Marta Canel, Morwenna Muir, Jennifer E. Ring, Eleni Maniati, Andrew H. Sims, Jonathan A. Pachter, Valerie G. Brunton, Nick Gilbert, Stephen M. Anderton, Robert J.B. Nibbs, and Margaret C. Frame

SUPPLEMENTAL EXPERIMENTAL PROCEDURES

Animal Procedures

All experiments involving animals were carried out in accordance with the UK Coordinating Committee on Cancer Research guidelines by approved protocol (Home Office Project Licence no. 60/4248).

Generation of FAK Nuclear Localization Mutant (FAK-NLS)

Point mutations were introduced into wild-type FAK (FAK-wt) at R177A, R178A, K190A, K191A, K216A, and K218A using site directed mutagenesis. Specifically, 20 ng of pWZL (Hygro) FAK-wt DNA template was mixed with 0.5 μM of each primer in PfuUltra Hotstart master mix (Stratagene) and subjected to PCR with cycle conditions as follows: 95°C (30 s), 18 cycles; 95°C (30 s), 50°C (1 min), 68°C (12 min), final incubation 4°C (infinite). Following PCR, samples were incubated with 10 units of DpnI restriction enzyme at 37°C for 1 hour. The resulting product was transformed into TOP10 chemically competent bacteria (Life Technologies) and plated onto ampicillin agar plates. Colonies were selected and expanded in L broth containing ampicillin overnight, DNA was isolated using a QIAprep DNA miniprep kit (Qiagen), and mutational status was confirmed by sequencing. Three rounds of mutagenesis were required to generate FAK-NLS (R177A, R178A, K190A, K191A, K216A, K218A). Primer sequences were as follows:

FAK R177A/R178A forward,
CTAGGTTGCCTTGAAATCGCGGCATCCTACGGAGAGATGAG;
FAK R177A/R178A reverse,
CTCATCTCTCCGTAGGATGCCGCGATTTCAAGGCAACCTAG;
FAK K190A/K191A forward,
GAGGCAATGCATTAGAGGCGGCATCCAACTATGAAGTGCTAG;
FAK K190A/K191A reverse,
CTAGCACTTCATAGTTGGATGCCGCCTCTAATGCATTGCCTC;
FAK K216A/K218A forward,
GAAGAGTTTGCTAGATTCAGTGGCGGCCGCAACACTACGAAAATTAATC;
FAK K216A/K218A reverse,
GATTAATTTTCGTAGTGTTGCGGCCGCCACTGAATCTAGCAAACTCTTC.

Cell Lines

Isolation and generation of the FAK SCC cell model is described in detail in Serrels et al. (Serrels et al., 2012). Briefly, SCCs were induced in K14*Cre*ER *FAK*$^{flox/flox}$ mice on the FVB background using the DMBA/TPA two-stage skin chemical carcinogenesis protocol and cells

isolated. Following treatment with 4-hydroxytamoxifen, a FAK-null (FAK-/-) cell clone was isolated, and retroviral transduction was used to stably re-express FAK-wt, FAK kinase-dead (FAK-kd), and FAK-NLS. Briefly, Phoenix Ecotropic cells were transfected with pWZL (Hygro) FAK constructs using Lipofectamine 2000 (Life Technologies) according to manufacturer's instructions. Twenty-four hours post-transfection, cell culture supernatant was removed, filtered through a 0.45-µm Millex-HA filter (Millipore), diluted at a 1:1 ratio in normal SCC cell culture medium, supplemented with 5 µg/ml polybrene, and added to SCC FAK-/- cells for 24 hours. A total of two rounds of infection were performed to generate each cell line. Cells were cultured at 37°C in Glasgow minimum essential medium (MEM) (Sigma-Aldrich) supplemented with 2 mM L-glutamine, MEM vitamins, 1 mM sodium pyruvate (all Sigma-Aldrich), MEM amino acids, and 10% fetal bovine serum (FBS) (both Life Technologies), and maintained under selection using 0.25 mg/ml hygromycin.

Western Blotting

To prepare whole cell lysates, cells were washed twice in ice-cold phosphate-buffered saline (PBS) and lysed in RIPA buffer [50 mM Tris-HCl (pH 7.6), 150 mM sodium chloride, 1% Triton X-100, 0.5% sodium deoxycholate, 0.1% SDS, cOmplete Ultra protease inhibitor cocktail (Roche), PhosSTOP phosphatase inhibitor cocktail (Roche)]. Cytoplasmic and nuclear extracts were prepared as described previously (Lim et al., 2008) and summarized below. Lysates were resolved by 4–15% Bis-Tris gel electrophoresis (Biorad), and proteins were transferred to nitrocellulose then blocked [5% bovine serum albumin in PBS–Tween 20 (BSA/PBS-T)] and probed with either anti-Bcl2 (Cell Signaling Technology), anti-FAK (Becton Dickinson), anti-PARP (Cell Signaling Technology), anti-GAPDH (Cell Signaling Technology), rabbit anti-TAF9 (Abcam), or anti-tubulin (Becton Dickinson) primary antibodies (all 1:1000 in 5% BSA/PBS-T). Bound antibody was detected by incubation with anti-rabbit or anti-mouse DyLight800-conjugated secondary antibody or HRP-conjugated secondary antibody (Cell Signaling Technology) and visualized using a Licor Odyssey imaging system or a BioRad ChemiDoc MP Imaging System.

Nuclear Fractionation

SCC FAK-wt and SCC FAK-/- cells were grown on tissue culture dishes for 48 hours at 37°C prior to fractionation. Keratinocyte cultures were prepared from two-day-old FVB pups as described previously (McLean et al., 2004). Keratinocytes pooled from seven pups were grown on two tissue culture dishes for 72 hours at 37°C prior to fractionation. For subcellular fractionation, cells were washed twice with ice-cold PBS, scraped into ice-cold cyto buffer [10 mM Tris-HCl (pH 7.5), 100 mM NaCl, 0.05% NP-40, 3 mM $MgCl_2$, 1 mM EGTA, cOmplete Ultra protease inhibitor cocktail (Roche), PhosSTOP phosphatase inhibitor cocktail

(Roche)] and incubated for 5 min on ice. Cytosolic supernatants were collected following centrifugation at 800 × *g* for 5 min at 4°C. Nuclear pellets were washed twice in cyto buffer, re-suspended in RIPA buffer and incubated for 15 min at 4°C. Nuclear supernatants were collected following centrifugation at 18,000 × *g* for 15 min at 4°C. For whole cell lysates, cells were washed twice with ice-cold PBS, scraped into RIPA buffer, and incubated for 15 min at 4°C. Clarified supernatants were collected by centrifugation at 18,000 × *g* for 15 min at 4°C. Protein concentration was determined by BCA protein assay (Thermo Fisher Scientific), and Western blot analysis performed as described above.

Subcutaneous Tumor Growth

Cells were injected subcutaneously into both flanks of either CD-1 nude mice (0.25×10^6 cells) or FVB mice (1×10^6 cells) and tumor growth measured twice-weekly using calipers. Animals were sacrificed when tumors reached maximum allowed size, or more commonly when signs of ulceration were evident. For studies involving treatment with VS-4718, drug was prepared in 0.5% carboxymethyl cellulose containing 0.1% Tween 80 (Sigma-Aldrich), and mice were treated at 75 mg/kg BID by oral gavage. Animals were visually monitored for signs of toxicity and weighed prior to each dose of VS-4718. No signs of toxicity were observed. Group sizes ranged from 3–5 mice, each bearing two tumors, and tumor volume was calculated in Excel (Microsoft) using the formula $v = 4/3\pi r^3$. Statistics and graphs were calculated using Prism (GraphPad).

Tumor Growth Following Re-challenge

1×10^6 SCC FAK-/- cells were injected subcutaneously into the left flank of FVB mice and tumor growth measured twice-weekly as described above. Following tumor regression, mice were left for 7 days before being challenged with 1×10^6 SCC FAK-wt or FAK-/- cells injected subcutaneously into the right flank. Control groups were injected subcutaneously into both flanks at day 28 using mice that had not been pre-challenged with SCC FAK-/- cells. Tumor volume was calculated as described above.

$CD4^+$, $CD8^+$, and $CD25^+$ T-cell Depletion

Anti-mouse CD4 (GK1.5, ATCC TIB-207) and CD8 (2.43, ATCC TIB-210) depleting antibodies were purified in-house from conditioned supernatant using a Melon Gel IgG purification kit or purchased from eBioscience (anti-CD4 clone GK1.5, anti-CD8 clone 53-6.7). Rat IgG isotype control and anti-mouse CD25 (clone PC61.5) antibodies were purchased from eBioscience. T-cell depletion was achieved following intra-peritoneal (IP) injection of 150 µg of depleting antibody (same for all antibodies) into female age-matched FVB mice for 3 consecutive days, and maintained by further IP injection at 3-day intervals

until the study was terminated. 1 × 10^6 SCC FAK-wt or FAK-/- cells were injected subcutaneously into both flanks 6 days after initial antibody treatment, and tumor growth was measured twice-weekly as described above. The extent of T-cell depletion was determined at the end of the study using FACS analysis of disaggregated spleen and thymus tissue (Figure S1).

FACS Analysis of Immune Cell Populations

Tumors established following injection of 1 × 10^6 SCC cells into both flanks of FVB mice were removed at day 7 into RPMI (Sigma-Aldrich) supplemented with 10% FBS (Life Technologies). Tumor tissue was mashed into a pulp using a scalpel and re-suspended in DMEM (Sigma-Aldrich) supplemented with 2 mg/ml collagenase D (Roche). Samples were incubated for 1 hour at 37°C, pelleted by centrifugation at 1600 rpm for 5 min at 4°C, re-suspended in 5 ml of red blood cell lysis buffer (Pharm Lysis Buffer, Becton Dickinson) for 5 min at 37°C, pelleted by centrifugation at 1600 rpm for 5 min at 4°C, re-suspended in PBS and passed through a 70-μm cell strainer. The resulting single cell suspension was pelleted by centrifugation at 1600 rpm for 5 min at 4°C and re-suspended in FACS buffer (PBS containing 1% FBS and 0.1% sodium azide). This step was repeated a total of three times. A sample of the suspension was counted using trypan blue exclusion and the remaining cell suspension pelleted by centrifugation at 1600 rpm for 5 min at 4°C and re-suspended in FACS buffer at a concentration of 1 × 10^6 viable cells/ml. One hundred microlitres of each sample were pipetted into each well of a 96-well plate and the plate centrifuged at 1600 rpm for 5 min at 4°C. Cell pellets were re-suspended in 50 μl of Fc block [1:200 dilution of Fc antibody (eBioscience) in FACS buffer] and incubated for 15 min at 4°C. Fifty microlitres of antibody mixture [1:200 dilution of antibodies (listed in Table S2) in FACS buffer] were added to each well and the samples incubated for 30 min in the dark at 4°C. The plate was then centrifuged at 1600 rpm for 5 min at 4°C and the cells re-suspended in FACS buffer and analyzed using a BD FACS Aria II. Regulatory T-cell staining was performed using a Treg staining kit (eBioscience). Staining of spleen and thymus tissue was performed as above. Data analysis was performed using FlowJo software. All antibodies were from eBioscience. For absolute counting, the same protocol as above was used, except tumors were weighed following surgical removal, and prior to FACS analysis samples were resuspended in varying volumes of FACS buffer (dependent on weight) containing CountBright Absolute Counting Beads (Life Technologies). Statistics and graphs were calculated using Prism (GraphPad).

Gene Expression Profiling

RNA was prepared from SCC FAK-wt and SCC FAK-/- cells using an RNAeasy kit (Qiagen) according to the manufacturer’s instructions. RNA was analysed using the GeneChip Mouse

Genome 430 2.0 Array (Affymetrix). Gene expression data were analyzed using packages within Bioconductor (Gentleman et al., 2004) implemented in the R statistical programming language. The gene expression data were summarised from CEL files using Ensembl gene identifiers and normalized using the Robust Multi-array Average algorithm (Wu and Irizarry, 2004) within the 'affy' package. The data discussed in this publication have been deposited in NCBI's Gene Expression Omnibus (Edgar et al., 2002) and are accessible through GEO Series accession number GSE71662. Data for differentially expressed genes ($p < 0.01$) were median centered and subjected to unsupervised agglomerative hierarchical clustering on the basis of Euclidean distance computed with a complete-linkage matrix using Cluster 3.0 (C Clustering Library, version 1.37) (de Hoon et al., 2004). Clustering results were visualized using Java TreeView (version 1.1.1) (Saldanha, 2004). Functional enrichment analysis against the mouse genome background was performed using ToppGene (Chen et al., 2009).

Quantitative RT^2-PCR Array Analysis of Cytokine, Chemokine, and Chemokine Receptor Expression

RNA prepared from SCC cells was analyzed using the mouse cytokine and chemokine RT^2 Profiler PCR Array (PAMM-150Z; Qiagen) and that from isolated Tregs (isolated using Treg isolation kit, 130-091-041; Miltenyi Biotech) was analyzed using the mouse chemokine and receptor array (PAMM-022Z; Qiagen) according to the manufacturer's instructions. Relative gene expression ($2^{-\Delta Ct}$) values were log transformed, median centered and subjected to hierarchical clustering as for microarray analysis. For interaction network analysis, an interactome of chemokine ligands and receptors was constructed using the IUPHAR/BPS Guide to Pharmacology database (Alexander et al., 2013; Pawson et al., 2014) and curated from the literature (Bachelerie et al., 2014), onto which expression data for detected genes were mapped and visualized using Cytoscape (version 3.0.2) (Shannon et al., 2003). In addition, expression of selected cytokine and chemokine genes was assessed by standard quantitative RT-PCR. TGFβ2-specific primers were purchased from Qiagen (catalog no. PPM02992A). Ccl5 primers used were: forward, CCCTCACCATCATCCTCACT; reverse, CCTTCGAGTGACAAACACGA. Cxcl10 primers used were: forward, CCCACGTGTTGAGATCATTG; reverse, CACTGGGTAAAGGGGAGTGA. B2M primers used were: forward, GGGAAGCCGAACATACTGAA; reverse, TGCTTAACTCTGCAGGCGTAT. Briefly, a reaction mix consisting of 10 µl SensiFAST SYBR Hi-ROX reagent (Bioline), 0.4 µl of 10 µM stock forward primer, 0.4 µl of 10 µM stock reverse primer, 4.4 µl water, and 4 µl of 20 ng/µl stock random hexamer primed cDNA was run on a Rotor-Gene qRT-PCR machine (Qiagen) using the following cycling conditions: 94°C for 10 min; 40 cycles of 94°C for 10 s, 57°C for 20 s, 72°C for 20 s; and 72°C for 7 min.

Analysis was performed using Rotor-Gene software, and expression relative to B2M was calculated using Excel (Microsoft).

shRNA-mediated TGFβ2 and Ccl5 Knockdown

To generate lentiviral particles, 2 × 10^6 HEK293FT cells were transfected with a mixture of 10 μg shRNA (RMM4534-EG21808; GE Healthcare), 6.5 μg HIV, and 3 μg VSVG plasmid DNA using Lipofectamine 2000 as per manufacturer's protocol. Twenty-four hours post-transfection, medium was removed and filtered through a 0.45-μm Millex-AC filter (Millipore) and mixed at a 1:1 ratio with normal SCC growth medium. This mixture was supplemented with polybrene to a final concentration of 5 μg/ml and added to SCC cells for 24 hours. Cells were subject to two rounds of lentiviral infection prior to selection with puromycin at a final concentration of 2 μg/ml. All shRNA constructs used were part of the pLKO lentiviral TRC library (GE Healthcare).

Preparation and Fractionation of Nuclei and Chromatin

Nuclei were prepared as previously described (Gilbert et al., 2003) but with a reduced concentration (0.05%) of NP-40 in nuclei buffer B. Soluble chromatin was prepared as described previously (Gilbert et al., 2004) and fractionated on a 5-ml sucrose step gradient to separate soluble and chromatin-associated nuclear proteins. Essentially, the soluble chromatin was layered over 10% and 50% sucrose in TEP80 buffer [10 mM Tris-HCl (pH 8.0), 1 mM EDTA, 80 mM NaCl, 100 μM PMSF] and centrifuged at 50,000 rpm for 1 h 50 min in an MLS-50 rotor (Beckman). Five hundred–microlitre fractions were collected by upward displacement in a gradient fractionator (Teledyne ISCO). DNA was recovered from fractions using a PCR purification kit (Qiagen) and subjected to agarose (1.5%) gel electrophoresis. Protein was purified using TCA precipitation. Specifically, one volume of TCA stock solution was mixed with four volumes of protein solution, incubated at 4°C for 10 min, and spun at 14,000 rpm for 5 min at 4°C. The supernatant was discarded, and the pellet was washed twice with ice-cold acetone and dried for 5 min at room temperature. Samples were analysed by SDS-PAGE and western blotted using anti-FAK (Cell Signalling Technologies), anti-HP1α (Millipore), and anti-histone H3 (Cell Signalling Technologies) antibodies.

Proteomic Analysis of Nuclear FAK Protein Complexes

Two milligrams of nuclear lysates were incubated with 10 μl agarose-conjugated anti-FAK antibody (clone 4.47; Merck Millipore) overnight at 4°C with rotation. Beads were washed twice with ice-cold RIPA buffer and twice with ice-cold PBS. Bead-bound complexes from biological triplicate experiments were subjected to on-bead proteolytic digestion, desalting

and liquid chromatography–tandem mass spectrometry in technical duplicate as described previously (Turriziani et al., 2014). Mean label-free MS intensities were calculated from technical duplicate data acquisitions for each biological replicate. Peptide and protein false discovery rates were set to 1%. Proteins enriched from SCC FAK-wt nuclei by at least two-fold over SCC FAK-/- control nuclei ($p < 0.05$) were considered specifically isolated by FAK immunoprecipitation. For interaction network analysis, Ccl5 transcription factors were extracted from the DECODE database (Qiagen) (most relevant transcription factors predicted to bind between 20 kb upstream and 10 kb downstream of the Ccl5 transcription starting site for human, mouse and rat) and used to seed a network of 1000 transcription factor–related proteins using the GeneMANIA plugin (version 2014-08-12-core; human interactions) in Cytoscape. Proteins specifically isolated in nuclear FAK protein complexes were mapped onto the interactome, and those with physical or predicted direct or indirect interactions with Ccl5 transcription factors were analyzed using the NetworkAnalyzer plugin (version 2.7) in Cytoscape.

CD8 T-cell Fluorescent Immunohistochemistry

SCC tumors were surgically removed 7 days post-implantation and snap-frozen in a cryovial by submersing in liquid nitrogen. Tumor sections were cut using a cryostat and stored at –80°C. Prior to staining, sections were moved to –20°C overnight. Slides were air-dried for 30 min, fixed with ice-cold acetone (Sigma-Aldrich) for 10 min at room temperature, and washed three times for 5 min each in PBS. Tumor sections were outlined using a hydrophobic barrier pen (DAKO), then incubated with mouse background block for 1 h at room temperature. Samples were incubated with primary antibody [anti-mouse CD8α (clone 53-6.7), rat IgG2a; R&D Systems; 1:100 dilution in 5% goat serum–2.5% BSA in PBS] for 1 h at room temperature, then washed three times with PBS-T, each for a period of 5 min, followed by three 5-min PBS washes. Samples were incubated with secondary antibody [Alexa Fluor 647 goat anti-rat IgG (H+L) (Life Technologies), 1:200 dilution] for 1 h at room temperature in the dark, then subjected to three 5-min washes with PBS-T, followed by one 5-min wash with PBS and one wash with sterile water. Samples were mounted under a coverslip (Thermo Scientific) using ProLong Gold Antifade Mount containing DAPI (Life Technologies) and imaged using an Olympus FV1000 confocal microscope. Images were acquired using an UPLSAPO 20× lens. DAPI and Alexa Fluor 647 were imaged using 405-nm and 635-nm excitation lasers. Three-dimensional image stitching was performed using the Olympus multi-site time-lapse module, with the final image comprising an 8 × 8 matrix with 20% overlap between adjacent images. Images were rendered using Imaris (Bitplane).

SUPPLEMENTAL TABLES

Table S2. Markers and Antibody Combinations Used to Define Immune Cell Populations, Related to Experimental Procedures

(A) T-cell subsets for FACS analysis. (B) Macrophage subsets for FACS analysis. (C) MDSC subsets for FACS analysis. (D) Treg subsets for FACS analysis.

A

Population	Markers
$CD4^+$ T cell	$CD45^+$ $CD3^+$ $CD4^+$ $CD8^-$
$CD4^+$ Central Memory T cell	$CD45^+$ $CD3^+$ $CD4^+$ $CD8^-$ $CD44^{hi}$ $CD62L^{hi}$
$CD4^+$ Effector T cell	$CD45^+$ $CD3^+$ $CD4^+$ $CD8^-$ $CD44^{hi}$ $CD62L^{low}$
$CD8^+$ T cell	$CD45^+$ $CD3^+$ $CD4^-$ $CD8^+$
$CD8^+$ Central Memory T cell	$CD45^+$ $CD3^+$ $CD4^-$ $CD8^+$ $CD44^{hi}$ $CD62L^{hi}$
$CD8^+$ Effector T cell	$CD45^+$ $CD3^+$ $CD4^-$ $CD8^+$ $CD44^{hi}$ $CD62L^{low}$

Marker	Fluorophore	Filter Settings
Viability	Viability 506	405_525/50
CD45	e450	405_450/50
CD3	FITC	488_525/50
CD8	PE	561_582/15
CD4	e647	640_617/14
CD62L	PE-Cy7	561_780/60
CD44	PerCP - Cy5.5	488_685/35

B

Population	Markers
Inflammatory Macrophage (iMac)	$CD45^+$ $CD11b^+$ $F4/80^+$ $Ly6C^+$
Tissue-Resident Macrophage (Mac)	$CD45^+$ $CD11b^+$ $F4/80^+$ $Ly6C^-$

Marker	Fluorophore	Filter Settings
Viability	Viability 506	405_525/50
CD45	e780	640_780/60
F4/80	FITC	488_525/50
CD11b	PerCP - Cy5.5	488_685/35
Ly6C	e450	405_450/50

C

Population	Markers
M-MDSC	$CD45^+$ $CD11b^+$ $F4/80^-$ $Ly6C^{hi}$ $Gr1^{lo}$
G-MDSC	$CD45^+$ $CD11b^+$ $F4/80^-$ $Ly6C^{int}$ $Gr1^{hi}$

Marker	Fluorophore	Filter Settings
Viability	Viability 506	405_525/50
CD45	e780	640_780/60
CD11b	PerCP - Cy5.5	488_685/35
F4/80	PE-Cy7	561_780/60
Ly6C	e450	405_450/50
Gr1	FITC	405_450/50

D

Population	Markers
Treg	$CD4^+$ $FoxP3^{hi}$ $CD25^{hi}$

Marker	Fluorophore	Filter Settings
CD4	PerCP - Cy5.5	488_685/35
CD25	PE	561-582_15
FoxP3	FITC	405_450/50

SUPPLEMENTAL REFERENCES

Alexander, S.P., Benson, H.E., Faccenda, E., Pawson, A.J., Sharman, J.L., McGrath, J.C., Catterall, W.A., Spedding, M., Peters, J.A., Harmar, A.J.; CGTP Collaborators, et al. (2013). The Concise Guide to PHARMACOLOGY 2013/14: overview. Br. J. Pharmacol. *170*, 1449–1458.

Bachelerie, F., Graham, G.J., Locati, M., Mantovani, A., Murphy, P.M., Nibbs, R., Rot, A., Sozzani, S., and Thelen, M. (2014). New nomenclature for atypical chemokine receptors. Nat. Immunol. *15*, 207–208.

Chen, J., Bardes, E.E., Aronow, B.J., and Jegga, A.G. (2009). ToppGene Suite for gene list enrichment analysis and candidate gene prioritization. Nucleic Acids Res. *37*, W305–W311.

de Hoon, M.J., Imoto, S., Nolan, J., and Miyano, S. (2004). Open source clustering software. Bioinformatics *20*, 1453–1454.

Edgar, R., Domrachev, M., and Lash, A.E. (2002). Gene Expression Omnibus: NCBI gene expression and hybridization array data repository. Nucleic Acids Res. *30*, 207–210.

Gentleman, R.C., Carey, V.J., Bates, D.M., Bolstad, B., Dettling, M., Dudoit, S., Ellis, B., Gautier, L., Ge, Y., Gentry, J., Hornik, K., Hothorn, T., Huber, W., Iacus, S., Irizarry, R., Leisch, F., Li, C., Maechler, M., Rossini, A.J., Sawitzki, G., Smith, C., Smyth, G., Tierney, L., Yang, J.Y., and Zhang, J. (2004). Bioconductor: open software development for computational biology and bioinformatics. Genome Biol. *5*, R80.

Pawson, A.J., Sharman, J.L., Benson, H.E., Faccenda, E., Alexander, S.P., Buneman, O.P., Davenport, A.P., McGrath, J.C., Peters, J.A., Southan, C., Spedding, M., Yu, W., Harmar, A.J.; NC-IUPHAR. (2014). The IUPHAR/BPS Guide to PHARMACOLOGY: an expert-driven knowledgebase of drug targets and their ligands. Nucleic Acids Res. *42*, D1098–D1106.

Saldanha, A.J. (2004). Java Treeview--extensible visualization of microarray data. Bioinformatics *20*, 3246–3248.

Shannon, P., Markiel, A., Ozier, O., Baliga, N.S., Wang, J.T., Ramage, D., Amin, N., Schwikowski, B., and Ideker, T. (2003). Cytoscape: a software environment for integrated models of biomolecular interaction networks. Genome Res. *13*, 2498–2504.

Wu, Z. and Irizarry, R.A. (2004). Preprocessing of oligonucleotide array data. Nat. Biotechnol. *22*, 656–658; author reply 658.
