# Supplementary material for: Nuclear FAK Controls Chemokine Transcription, Tregs, and Evasion of Anti-tumor Immunity
Source: Cell. 2015 Sep 24;163(1):160–73. doi: 10.1016/j.cell.2015.09.001 (PMC4597190; doi:10.1016/j.cell.2015.09.001)
Supplement: Document S2. Article plus Supplemental Information [file mmc3.pdf]

Cell

Article

Nuclear FAK Controls Chemokine Transcription, Tregs, and Evasion of Anti-tumor Immunity

Graphical Abstract

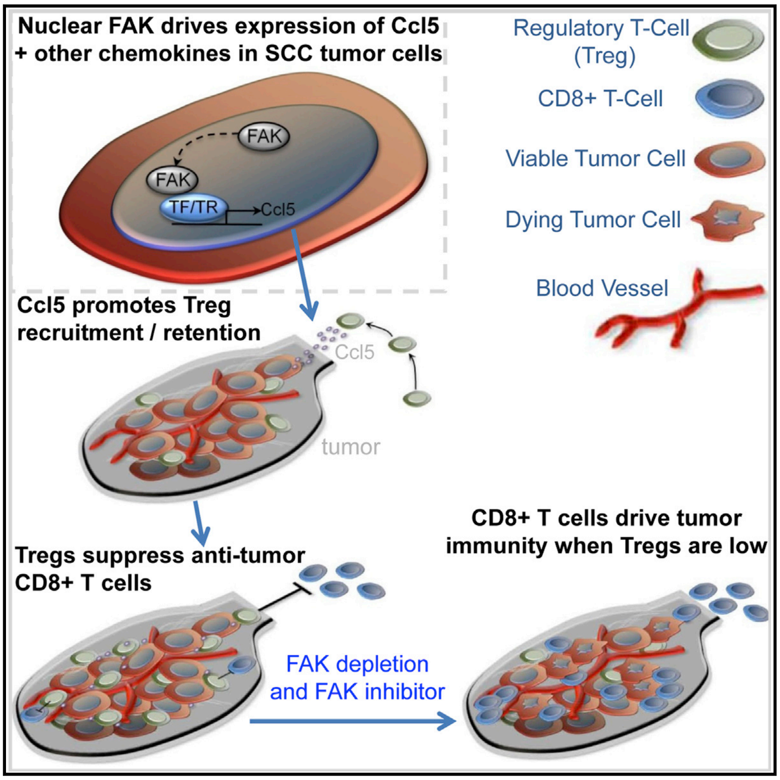

Highlights

- Depletion or kinase inhibition of FAK can cause squamous cell carcinoma regression
- FAK promotes tumor evasion by inducing an immuno-suppressive microenvironment
- Nuclear FAK promotes transcription of chemokines that drive recruitment of Tregs
- FAK-induced Tregs inhibit cytotoxic CD8+ T cells, allowing tumor tolerance and growth

Authors

Alan Serrels, Tom Lund, Bryan Serrels, ..., Stephen M. Anderton, Robert J.B. Nibbs, Margaret C. Frame

Correspondence

a.serrels@ed.ac.uk (A.S.),
m.frame@ed.ac.uk (M.C.F.)

In Brief

Nuclear focal adhesion kinase (FAK) regulates transcription of chemokines that drive recruitment of tumor-associated regulatory T cells (Tregs), thereby creating a tumor suppressive microenvironment by inhibiting cytotoxic CD8+ T cell activity.

Accession Numbers

GSE71662

CrossMark

Serrels et al., 2015, Cell *163*, 160–173
September 24, 2015

©2015 The Authors

http://dx.doi.org/10.1016/j.cell.2015.09.001

CellPress

Article

Nuclear FAK Controls Chemokine Transcription, Tregs, and Evasion of Anti-tumor Immunity

Alan Serrels,[1,7,*] Tom Lund,[1,7] Bryan Serrels,[1] Adam Byron,[1] Rhoanne C. McPherson,[2] Alexander von Kriegsheim,[1] Laura Gómez-Cuadrado,[1] Marta Canel,[1] Morwenna Muir,[1] Jennifer E. Ring,[3] Eleni Maniati,[4] Andrew H. Sims,[1] Jonathan A. Pachter,[3] Valerie G. Brunton,[1] Nick Gilbert,[5] Stephen M. Anderton,[2] Robert J.B. Nibbs,[6] and Margaret C. Frame[1,*]

[1]Edinburgh Cancer Research UK Centre, Institute of Genetics and Molecular Medicine, University of Edinburgh, Edinburgh EH4 2XR, UK
[2]MRC Centre for Inflammation Research, The Queens Medical Research Institute, University of Edinburgh, Edinburgh EH16 4TJ, UK
[3]Verastem Inc., 117 Kendrick Street, Suite 500, Needham, MA 02494, USA
[4]Queen Mary, University of London, Centre for Cancer and Inflammation, Charterhouse Square, London EC1M 6BQ, UK
[5]MRC Human Genetics Unit, Institute of Genetics and Molecular Medicine, University of Edinburgh, Edinburgh EH4 2XU, UK
[6]Institute of Infection, Immunity, and Inflammation, University of Glasgow, Glasgow G12 8TA, UK
[7]Co-first author
*Correspondence: a.serrels@ed.ac.uk (A.S.), m.frame@ed.ac.uk (M.C.F.)
http://dx.doi.org/10.1016/j.cell.2015.09.001
This is an open access article under the CC BY license (http://creativecommons.org/licenses/by/4.0/).

SUMMARY

Focal adhesion kinase (FAK) promotes anti-tumor immune evasion. Specifically, the kinase activity of nuclear-targeted FAK in squamous cell carcinoma (SCC) cells drives exhaustion of CD8⁺ T cells and recruitment of regulatory T cells (Tregs) in the tumor microenvironment by regulating chemokine/cytokine and ligand-receptor networks, including via transcription of Ccl5, which is crucial. These changes inhibit antigen-primed cytotoxic CD8⁺ T cell activity, permitting growth of FAK-expressing tumors. Mechanistically, nuclear FAK is associated with chromatin and exists in complex with transcription factors and their upstream regulators that control Ccl5 expression. Furthermore, FAK's immuno-modulatory nuclear activities may be specific to cancerous squamous epithelial cells, as normal keratinocytes do not have nuclear FAK. Finally, we show that a small-molecule FAK kinase inhibitor, VS-4718, which is currently in clinical development, also drives depletion of Tregs and promotes a CD8⁺ T cell-mediated anti-tumor response. Therefore, FAK inhibitors may trigger immune-mediated tumor regression, providing previously unrecognized therapeutic opportunities.

INTRODUCTION

First described more than a decade ago (Onizuka et al., 1999; Shimizu et al., 1999), regulatory T cells (Tregs) have become recognized as a core component of the immuno-suppressive armory utilized by many tumors to keep the anti-tumor activity of antigen-primed CD8⁺ T cells at bay. Increased Treg numbers has been associated with poorer survival in ovarian (Curiel et al., 2004), gastrointestinal (Sasada et al., 2003), and esophageal (Kono et al., 2006) cancer. Indeed, the ratio of CD8⁺ T cells/Tregs correlates with poor prognosis, shifting the balance from anti-tumor immunity toward tumor tolerance (Quezada et al., 2006; Sato et al., 2005; Shah et al., 2011). Through secreting a range of chemokines and cytokines, cancer cells can promote the recruitment of Tregs into tumors and can also facilitate their peripheral expansion and retention (Darrasse-Jèze and Podsypanina, 2013; Ondondo et al., 2013). Thus, Tregs can act as a barrier to effective immune-based therapy aimed at activation of a CD8⁺ T cell anti-tumor immune response. However, the specific signals within tumor cells that stimulate elevated intra-tumoral Tregs, giving rise to tumor tolerance, remain elusive.

FAK is a tyrosine kinase that regulates diverse cellular functions, including adhesion, migration, invasion, polarity, proliferation, and survival (Frame et al., 2010). Using targeted gene deletion in mouse skin, we have previously shown a requirement for *fak* in tumor initiation and progression to malignant disease (McLean et al., 2004). FAK is also required for mammary tumor progression, intestinal tumorigenesis, and the androgen-independent formation of neuroendocrine carcinoma in a mouse model of prostate cancer (Ashton et al., 2010; Lahlou et al., 2007; Luo et al., 2009a; Provenzano et al., 2008; Pylayeva et al., 2009; Slack-Davis et al., 2009). Expression of FAK is elevated in a number of tumor types (reviewed in McLean et al., 2005), and FAK inhibitors are being developed as potential cancer therapeutics (Roberts et al., 2008; Shapiro et al., 2014). Many of FAK's functions in cancer are via its role in signaling downstream of integrins and growth factor receptors at the plasma membrane. FAK also contains putative nuclear localization sequences (NLS) within the F2 lobe of its FERM domain and can localize to the nucleus upon receipt of cellular stress, where it binds to p53 (Lim et al., 2008). However, the extent of FAK's nuclear functions remains largely unknown. Here, we report a function for nuclear FAK in regulating transcription of inflammatory cytokines and chemokines, in turn promoting an immuno-suppressive, pro-tumorigenic microenvironment. This is mediated

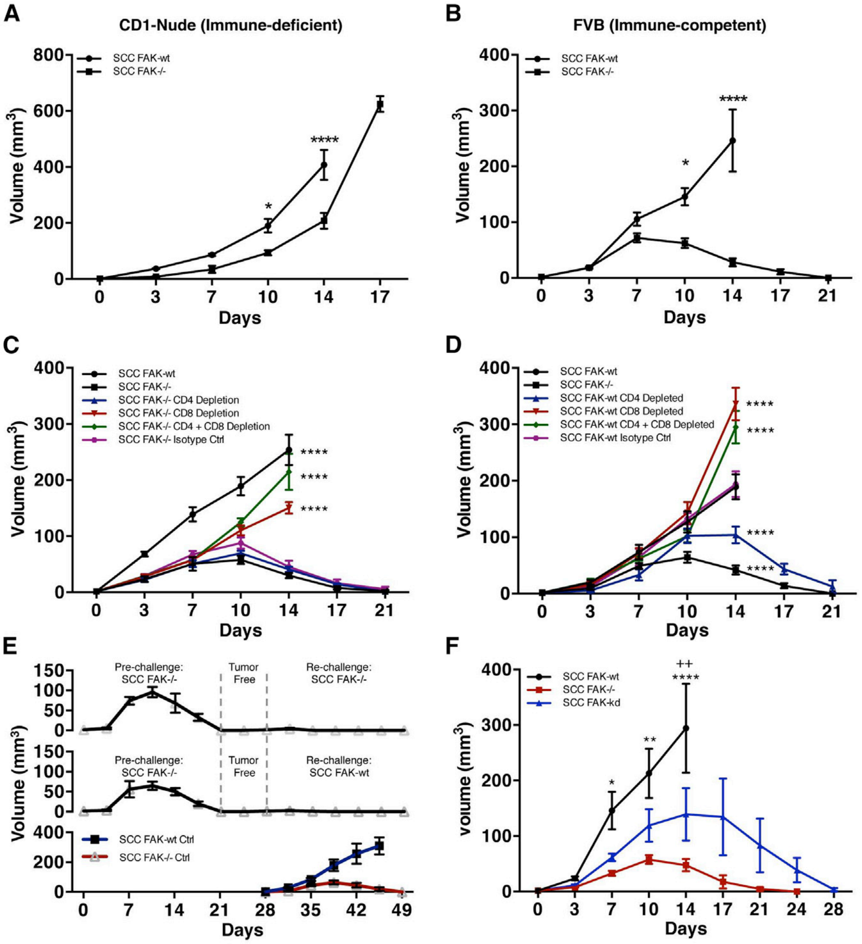

Figure 1. Loss of FAK or FAK Kinase Activity Results in CD8$^+$ T Cell-Dependent SCC Tumor Clearance

(A and B) SCC FAK-WT and SCC $FAK^{-/-}$ subcutaneous tumor growth in immune-deficient CD-1 nude mice (A) and immune-competent FVB mice (B).

(C and D) SCC $FAK^{-/-}$ (C) and SCC FAK-WT (D) tumor growth in FVB mice treated with T-cell-depleting antibodies.

(E) Secondary tumor re-challenge with SCC $FAK^{-/-}$ (top) and SCC FAK-WT (middle) cells following a pre-challenge with SCC $FAK^{-/-}$ cells and a 7-day tumor-free period. Subcutaneous growth of SCC FAK-WT and SCC $FAK^{-/-}$ tumors injected at day 28 without pre-challenge (bottom).

(F) Tumor growth in FVB mice following subcutaneous injection of SCC FAK-WT, SCC $FAK^{-/-}$, and SCC FAK-KD cells.

*$p < 0.05$, **p or $^{++}p < 0.01$, ****$p < 0.0001$; Sidak-corrected two-way ANOVA (A and B) or Tukey-corrected two-way ANOVA (C, versus SCC $FAK^{-/-}$; D, versus SCC FAK-WT; F, *, versus SCC $FAK^{-/-}$ and $^+$, versus SCC FAK-KD). Data are represented as mean ± SEM; n = 5–6 tumors.

by recruitment and expansion of Tregs via FAK-regulated chemokine/cytokine networks, and we have found an important role for Ccl5 and TGFβ2. Therefore, FAK controls the tumor environment, and suppressing FAK activity, including via a clinically relevant FAK inhibitor, may be therapeutically beneficial by triggering immune-mediated tumor regression.

RESULTS

FAK-Deficient SCC Tumors Undergo Regression in an Immune-Competent Host

We used a syngeneic model of SCC in which the *fak* gene had been deleted by Cre-lox recombination (McLean et al., 2004; Serrels et al., 2012) and mutant tumor cell lines generated. We monitored tumor growth following injection of 1 × 10^6 FAK-deficient cells ($FAK^{-/-}$) or FAK-deficient cells that re-expressed wild-type FAK (FAK-WT) at comparable levels to endogenous FAK in both CD-1 nude and FVB (syngeneic) host mouse strains. In CD-1 nude mice, SCC $FAK^{-/-}$ tumor growth was characterized by a modest growth delay (Figure 1A) as reported previously (Serrels et al., 2012). By contrast, in FVB mice, SCC $FAK^{-/-}$ tumor growth was characterized by an initial period of growth in the first 7 days followed by complete regression by day 21 (Figure 1B). Thus, FAK expression is required for the survival and growth of SCC tumors in FVB mice with a functional adaptive immune system.

SCC $FAK^{-/-}$ Tumor Regression Is Dependent on CD8$^+$ T Cells

To characterize the role of adaptive immunity in $FAK^{-/-}$ SCC tumor regression, we used antibody-mediated T cell depletion in animals bearing $FAK^{-/-}$ tumors (Figures 1C and S1). Depletion of CD4$^+$ T cells had no effect on tumor growth. In contrast, depletion of CD8$^+$ T cells, either alone or in combination with CD4$^+$ T cells, restored SCC $FAK^{-/-}$ tumor growth. This implies that cytotoxic CD8$^+$ T cells were responsible for regression of $FAK^{-/-}$ tumors (Figure 1C) but does not exclude an accessory role for CD4$^+$ T cells. T cell depletion in mice bearing SCC FAK-WT tumors (Figure 1D) revealed that: (1) depletion of CD8$^+$ T cells, either alone or in combination with CD4$^+$ T cells, caused a significant increase in tumor growth when compared to isotype-treated controls at day 14, and (2) depletion of CD4$^+$ T cells alone caused regression of FAK-WT SCC tumors by day 21. This implied that FAK-expressing tumors were also under negative pressure from the immune system and that cells from the CD4$^+$ T cell compartment play a role in protecting FAK-WT tumors from immune-mediated regression (reason discussed later; Figure 3).

Next, we re-challenged mice with 1 × 10^6 SCC FAK-WT cells after regression of primary $FAK^{-/-}$ SCC tumors, following 7 days of tumor-free survival after the tumors had regressed (Figure 1E, top and middle graphs). Neither FAK-deficient nor FAK-expressing SCC cells were able to grow after the mice had been pre-challenged with SCC $FAK^{-/-}$ cells. As controls, SCC FAK-WT and $FAK^{-/-}$ cells were injected at day 28 into mice with no pre-challenge, and these grew as expected (Figure 1E, bottom).

This implies that, following $FAK^{-/-}$ SCC tumor regression, host mice remain immunized against further tumor challenge because immunological memory had been established. It is possible that either broad immunization against SCCs may have occurred or, more likely, that the $FAK^{-/-}$ and FAK-WT SCCs shared common antigen(s) that are expressed irrespective of FAK status. We conclude that FAK enables SCC cancer cells to suppress an adaptive immune response rather than to circumvent it through evading recognition per se. SCC $FAK^{-/-}$ cells in which a FAK kinase-deficient mutant was re-expressed (SCC FAK-KD) initially grew and then regressed with kinetics that were only modestly delayed when compared to $FAK^{-/-}$ cells, indicating that immune suppression depends on FAK kinase activity (Figure 1F).

We next investigated the nature of the T cell response within tumors derived from all three SCC cell lines using FACS analysis on disaggregated tumor tissue taken at day 7. We did not observe a significant change in the percentage of total $CD4^+$ T cells (Figures 2A and S2 and Table S2) or the percentage of $CD4^+$ T cells that expressed the activation marker CD69 (Figure 2B). In contrast, we did observe a significant increase in the proportion of effector $CD4^+CD44^{hi}CD62L^{low}$ T cells in SCC $FAK^{-/-}$ and FAK-KD tumors when compared to FAK-WT tumors (Figures 2C and S2 and Table S2). Analysis of tumor-infiltrating $CD8^+$ T cells revealed a significant increase in SCC $FAK^{-/-}$ and SCC FAK-KD tumors when compared to SCC FAK-WT tumors (Figures 2D and S2 and Table S2), indicative of a heightened cytotoxic anti-tumor immune response. Staining with the activation marker CD69 identified the presence of $CD8^+CD69^+$ T cells in all tumors (Figure 2E). Further analysis revealed an increase in percentage of effector $CD8^+CD44^{hi}CD62L^{low}$ T cells in SCC $FAK^{-/-}$ and SCC FAK-KD tumors when compared to SCC FAK-WT tumors (Figures 2F and S2 and Table S2), especially when effector $CD8^+$ T cell numbers were normalized to account for the observed changes in total $CD8^+$ T cells and presented as a "fold change" (Figure 2G). However, while SCC $FAK^{-/-}$ and SCC FAK-KD tumors had increased effector $CD8^+$ T cells, there were activated $CD8^+$ T cells present in all of the SCC tumors, raising the question of why SCC FAK-WT tumors do not succumb to the cytotoxic $CD8^+$ T cell response.

It is now established that not only the quantity of tumor-infiltrating $CD8^+$ T cells is important, but also their "quality." Tumor-induced T cell exhaustion has been reported in a number of tumor types, including melanoma (Fourcade et al., 2010) and ovarian cancer (Matsuzaki et al., 2010), and is characterized by expression of co-inhibitory surface receptors, including programmed death receptor 1 (PD-1), lymphocyte-activation gene 3 (LAG-3), and T cell immunoglobulin mucin-3 (Tim-3), either alone or in combination (Fourcade et al., 2010; Sakuishi et al., 2010; Wherry, 2011). Analysis of these markers on antigen-primed $CD8^+CD44^{hi}$ T cells infiltrating SCC FAK-WT, $FAK^{-/-}$, and FAK-KD tumors revealed increased surface expression of PD-1, LAG-3, and Tim-3 in $CD8^+CD44^{hi}$ T cells present in SCC FAK-WT tumors (Figures 2H–2J). Together, our data imply that antigen-primed $CD8^+CD44^{hi}$ T cells infiltrating SCC FAK-WT tumors exhibit a heightened state of exhaustion indicative of a dysfunctional T cell response. Linked to their exhausted state, there was also evidence of decreased proliferation of $CD8^+$ T cells isolated from SCC FAK-WT tumors (judged by Ki-67 staining in Figure 2K).

Histological staining of tumor sections taken at day 7 revealed that: (1) $CD8^+$ T cells are present throughout all tumors, and (2) while $CD8^+$ T cells infiltrating SCC FAK-WT tumors appear predominantly as individual cells, $CD8^+$ T cells infiltrating SCC $FAK^{-/-}$ and FAK-KD tumors are clustered (Figure 2L). Thus, the ability of SCC FAK-WT tumors to evade the anti-tumor immune response is not due to limited $CD8^+$ T cell penetration into these tumors.

FAK Expression Drives Establishment of an Immuno-Suppressive Environment

Macrophages, myeloid-derived suppressor cells (MDSC), and Tregs with intrinsic immuno-suppressive capabilities can promote tumor development by inhibiting cytotoxic $CD8^+$ T cell activity in mouse and humans (Beyer and Schultze, 2006; Biragyn and Longo, 2012; Marigo et al., 2008). Flow cytometric analysis revealed no differences in macrophage or MDSC populations that correlated with tumor regression (Figures 3A, 3B, S3, and S4 and Table S2), although this does not rule out an accessory role for these cells in eventual tumor clearance. However, we did find a significantly greater number of $CD4^+FoxP3^+CD25^+$ Tregs in SCC FAK-WT tumors (Figures 3C and S4 and Table S2) when compared with $FAK^{-/-}$ and FAK-KD tumors (Figure 3C). Tregs have been associated with the development of $CD8^+$ T cell exhaustion (Sakuishi et al., 2013) and may therefore be linked to the $CD8^+$ T cell exhaustion that we observed in SCC FAK-WT tumors (Figures 2H–2J). We next calculated the ratio of $CD8^+$ T cells to Tregs (Figure 3D), as this has been reported to correlate with poor prognosis in a number of tumor types (Sato et al., 2005; Shah et al., 2011). We found a substantially lower $CD8^+$ T cell to Treg ratio in SCC FAK-WT tumors when compared to SCC $FAK^{-/-}$ and SCC FAK-KD tumors, which correlated with outcome in terms of tumor tolerance versus immune-mediated tumor regression.

Tregs Protect FAK-WT Tumors from Immune-Mediated Regression

We next examined SCC FAK-WT tumor growth in animals treated with an anti-CD25 antibody to deplete Tregs (Figure 3E). Depletion of $CD25^+$ cells led to regression of SCC FAK-WT tumors. Therefore, FAK-dependent Tregs are required for the growth of FAK-WT-expressing tumors by creating an immuno-suppressive environment that impairs cytotoxic $CD8^+$ T cell activity. This role of $CD4^+$ Tregs is the likely reason for effects of the CD4-depleting antibody in promoting regression of SCC FAK-WT tumors (Figure 1D). We note that high Treg levels have been reported in a number of solid tumor types (Beyer and Schultze, 2006) and that elevated Tregs are linked to poor clinical outcome (Beyer and Schultze, 2006; Sato et al., 2005).

We demonstrated that Tregs derived from SCC FAK-WT tumors expressed the transcription factor (TF) Helios (Figure S5A), indicative of thymic origin (Thornton et al., 2010). Thus, we hypothesized that FAK may drive the recruitment and expansion of the intra-tumoral Tregs by influencing the availability of secreted factors.

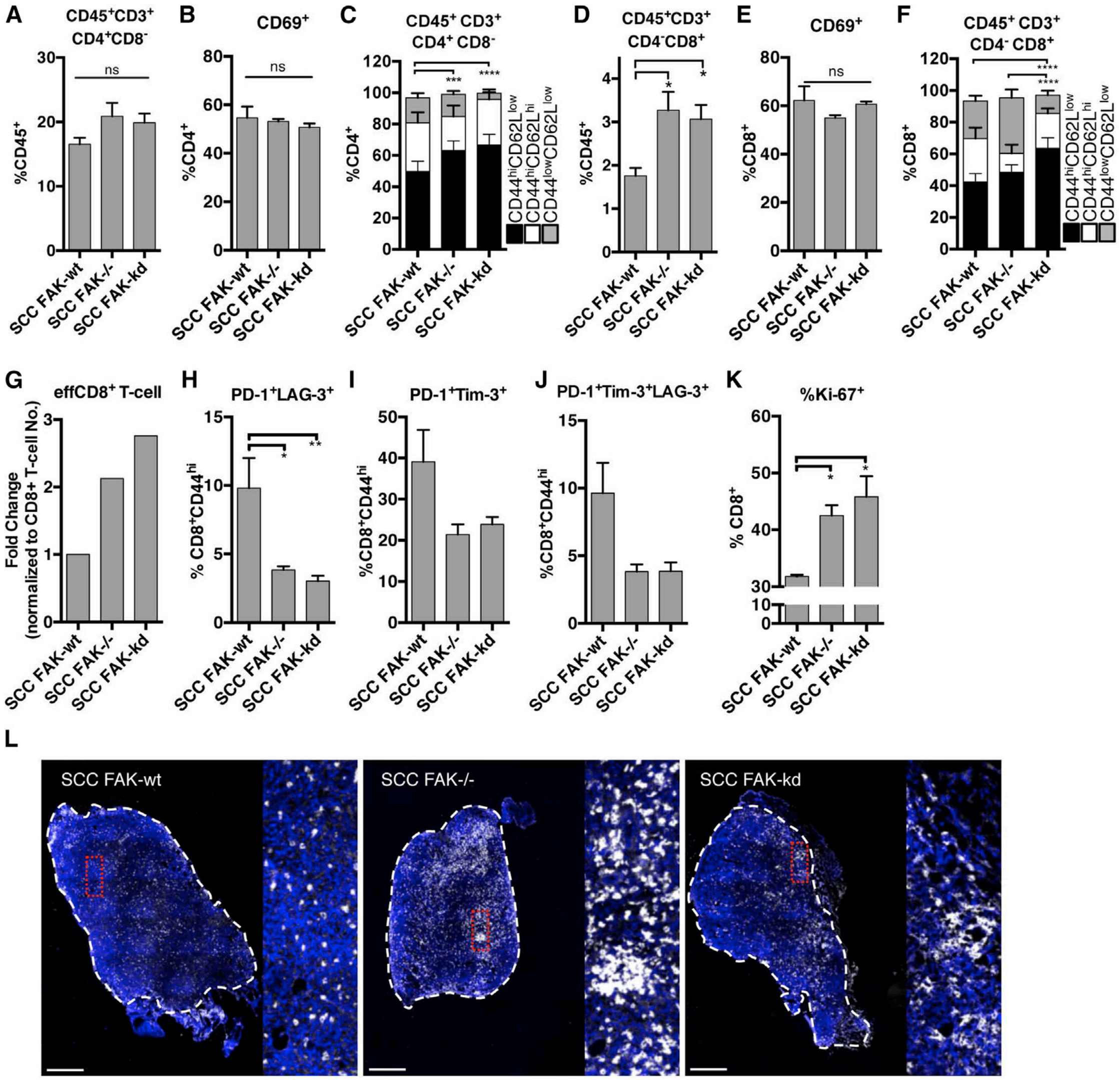

Figure 2. FAK-Depleted Tumors Exhibit a Heightened CD8+ T Cell Response

(A) FACS quantification of total intra-tumoral $CD4^+$ T cells.

(B) FACS quantification of $CD69^+$ cells as a percentage of $CD4^+$ T cells.

(C) FACS quantification of $CD4^+CD44^{hi}CD62L^{low}$, $CD4^+CD44^{hi}CD62L^{hi}$, $CD4^+CD44^{low}CD62L^{low}$ T cell subpopulations.

(D) FACS quantification of total intra-tumoral $CD8^+$ T cells.

(E) FACS quantification of $CD69^+$ cells as a percentage of $CD8^+$ T cells.

(F) Quantification of $CD8^+CD44^{hi}CD62L^{low}$, $CD8^+CD44^{hi}CD62L^{hi}$, $CD8^+CD44^{low}CD62L^{low}$ T cell subpopulations.

(G) Changes in effector ($CD8^+CD44^{hi}CD62L^{low}$) $CD8^+$ T cells normalized to total $CD8^+$ T cell proportions.

(H) FACS quantification of PD-1^+LAG-3^+ T cells as a percentage of $CD8^+CD44^{hi}$ tumor-infiltrating T cells. n = 6 tumors.

(I) FACS quantification of PD-1^+Tim-3^+ T cells as a percentage of $CD8^+CD44^{hi}$ tumor-infiltrating T cells. n = 3 tumors.

(J) FACS quantification of PD-1^+Tim-3^+LAG-3^+ T cells as a percentage of $CD8^+CD44^{hi}$ tumor-infiltrating T cells. n = 3 tumors.

(K) FACS quantification of Ki-67^+ cells as a percentage of tumor-infiltrating $CD8^+$ T cells. n = 3 tumors.

(L) Representative histological staining of CD8 in frozen sections from SCC FAK-WT, SCC $FAK^{-/-}$, and SCC FAK-KD tumors. Dashed white lines demark tumor boundary.

Scale bars, 500 μm. *$p < 0.05$, **$p < 0.01$, ***$p < 0.001$, ****$p < 0.0001$; ns, not significant; Tukey-corrected one-way ANOVA (C and F, $CD44^{hi}CD62L^{low}$ only). Data are represented as mean ± SEM; n = 5 tumors unless stated.

FAK Regulates the Transcription of Chemokines and Cytokines to Control Tregs

To address how FAK activity in SCC cancer cells promotes elevated intra-tumoral Tregs, we next analyzed global transcriptional profiles of SCC FAK-WT and SCC $FAK^{-/-}$ cells using Affymetrix GeneChip microarrays (Figure 4A). FAK expression resulted in the upregulation of 498 genes and the downregulation of 598 genes ($p < 0.01$). The upregulated transcript set in SCC

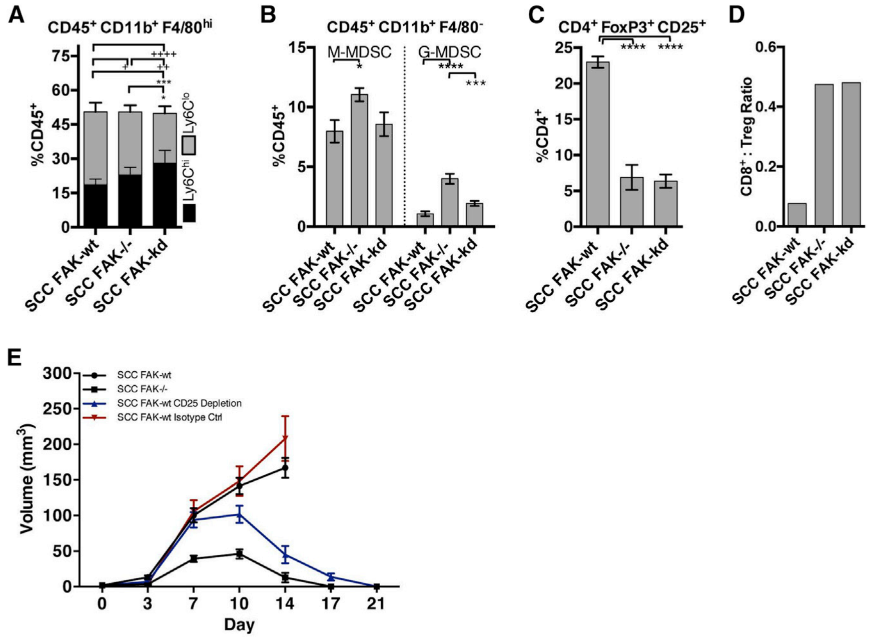

Figure 3. FAK Regulates the Immuno-Suppressive Microenvironment

(A) FACS quantification of Ly6C^{hi} and Ly6C^{low} macrophage populations expressed as a percentage of tumor-infiltrating CD45^{+} leukocytes.

(B) FACS quantification of Ly6C^{hi}Gr1^{low} (M-MDSC) and Ly6C^{int}Gr1^{hi} (G-MDSC) populations expressed as a percentage of tumor-infiltrating CD45^{+} leukocytes.

(C) Quantification of CD4^{+}CD25^{+}FoxP3^{+} Tregs expressed as a percentage of tumor-infiltrating CD4^{+} T cells.

(D) CD8^{+} T cell-to-Treg ratio calculated using mean values from Figures 2D and 3C.

(E) SCC FAK-WT tumor growth in FVB mice treated with anti-CD25 depleting antibody.

n = 6 tumors. * or $^{+}$p < 0.05, $^{++}$p < 0.01, ***p < 0.001, **** or $^{++++}$p < 0.0001; Tukey-corrected one-way ANOVA (A, *, Ly6C^{hi}; $^{+}$, Ly6C^{low}). Data are represented as mean ± SEM; n = 5 tumors unless stated.

FAK-WT cells was associated with a number of processes, including cell migration, receptor binding, secretion, wounding, and ovulation (Figure 4B, top). Analysis of this gene set revealed the chemokine ligand group of genes to be significantly overrepresented (Figure 4B, bottom), which is interesting given that a number of these chemokines and cytokines mediate both Treg recruitment to tumors and induction of peripheral Tregs within tumors (Goldstein et al., 2013; Ondondo et al., 2013).

To establish which chemokines and cytokines were regulated by FAK and to address whether the FAK-dependent transcriptional profile was linked to chemokine receptor expression on tumor-infiltrating Tregs, we performed quantitative (q)RT-PCR array analysis. Comparison of chemokine/cytokine transcript levels between SCC FAK-WT and SCC $FAK^{-/-}$ cells revealed a subset of ligands increased >2-fold in SCC FAK-WT cells (Figure 4C). Several of these (*Ccl1*, *Ccl5*, *Ccl7*, *Cxcl10*) have roles in Treg recruitment (Ondondo et al., 2013) (green arrowheads, Figure 4C), while one (*Tgfb2*) has a reported role in peripheral induction and expansion of Tregs (Goldstein et al., 2013) (red arrowhead, Figure 4C). To complement this, comparison of Tregs isolated from the thymus of normal FVB mice with those isolated directly from SCC FAK-WT tumors revealed a chemokine receptor switch (Figure 4D). We found increased expression of the cognate receptors for five of the six chemokine ligands upregulated in SCC FAK-WT cells (Figure 4C). These receptor changes may represent a switch from lymphoid homing receptors, including Ccr7 and Cxcr4, toward expression of memory/effector-type chemokine receptors, including Ccr2, Ccr5, Ccr8, and Cxcr6, involved in recruitment to non-lymphoid tissues and sites of inflammation. Network analysis of the relationship between FAK-dependent chemokine ligand expression in SCC cells and tumor-infiltrating Treg chemokine receptor expression revealed the existence of a FAK-dependent paracrine signaling axis between cancer cells and intra-tumoral Tregs based on chemokine ligand-receptor interactions (Figure 4E). Furthermore, (q)RT-PCR analysis of *Ccl5*, *Cxcl10*, and *Tgfb2* demonstrated that their expression was dependent on FAK kinase activity (Figure 4F). We note that disruption of the Ccl5/Ccr5 axis in a model of pancreatic adenocarcinoma results in reduced intra-tumoral Tregs and slows tumor growth (Tan et al., 2009), implying that FAK-dependent regulation of this paracrine signaling axis may be more generally important. Thus, FAK activity regulates the expression of a subset of chemokines that can specifically mediate crosstalk between tumor cells and tumor-infiltrating Tregs. This likely has importance in recruitment and retention of CD4^{+}FoxP3^{+}CD25^{+} Tregs into SCC FAK-WT tumors.

Nuclear FAK Regulates the Transcription of Ccl5 and TGFβ2 to Increase Tregs

The finding that the Tregs enriched in SCC FAK-WT tumors were likely recruited into SCC FAK-WT tumors led us to consider a potential role for Ccl5 that has been implicated in the recruitment and expansion of CD4^{+}FoxP3^{+}CD25^{+} Tregs (Tan et al., 2009), via the paracrine signaling axis that we identified. We found that efficient knockdown of *Ccl5* using two independent shRNA hairpins (P1 and P2, Figure 5A) resulted in SCC FAK-WT shRNA-Ccl5 tumor regression by days 21–27 (Figure 5B). We measured the absolute number of Tregs in SCC FAK-WT shRNA-Ccl5 tumors at day 7 and found that there was a substantial reduction in both Ccl5-depleted tumors when compared with empty vector control SCC FAK-WT pLKO tumors (Figure 5C).

Expanding on these findings, shRNA-mediated knockdown of *Tgfb2* expression in SCC FAK-WT cells also influenced tumor growth (Figures S5B and S5C). Partial knockdown of TGFβ2 had complex effects, which resulted in one of two outcomes. One group (Figure S5C, dashed blue line), grew more rapidly and ulcerated, leading to removal from study at day 14. In the

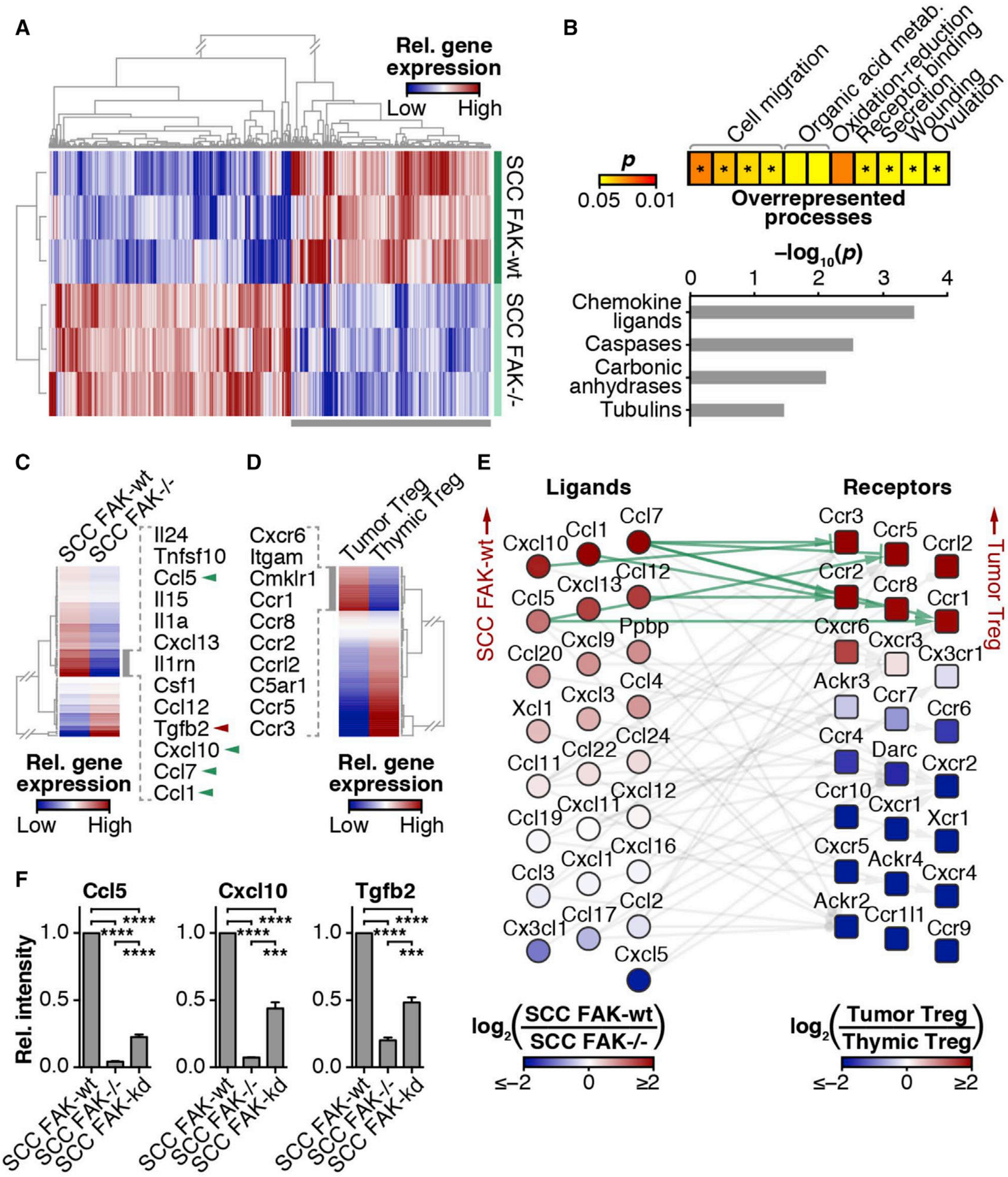

Figure 4. FAK Regulates Transcription of Cytokines Implicated in Treg Recruitment and Expansion

(A) Transcriptomic profiling of SCC FAK-WT and SCC *FAK*$^{-/-}$ cells.

(B) Functional enrichment analysis of genes upregulated in SCC FAK-WT cells (bottom gray bar in A). Overrepresented biological processes are displayed as a heatmap (log_{10}-transformed color scale) (top); asterisks indicate presence of cytokine-related genes. Overrepresented gene families are displayed as a bar chart (bottom). $p < 0.05$; Benjamini–Hochberg-corrected hypergeometric tests.

(C) qRT-PCR array analysis of cytokine and chemokine expression in SCC FAK-WT and SCC *FAK*$^{-/-}$ cells. Gray bar indicates cluster of genes upregulated in SCC FAK-WT cells; cytokine and chemokine gene names are listed. Green arrowheads indicate reported roles in Treg recruitment; red arrowhead indicates reported role in peripheral Treg induction.

(D) qRT-PCR array analysis of chemokine and receptor expression in tumor- and thymus-derived Tregs. Gray bar indicates cluster of genes upregulated in tumor-derived Tregs; receptor gene names are listed.

(E) Interaction network analysis of chemokine ligand gene expression detected in SCC cells (circles, left) and corresponding receptor gene expression detected in Tregs (squares, right). Genes are ordered vertically by fold change. Light gray lines connect receptor-ligand pairs; green lines indicate pairs upregulated at least 2-fold in SCC FAK-WT cells and tumor-derived Tregs.

(F) qRT-PCR analysis of selected cytokine and chemokine gene expression in SCC cells. ***$p < 0.001$, ****$p < 0.0001$; Tukey-corrected one-way ANOVA. Data are represented as mean ± SEM.

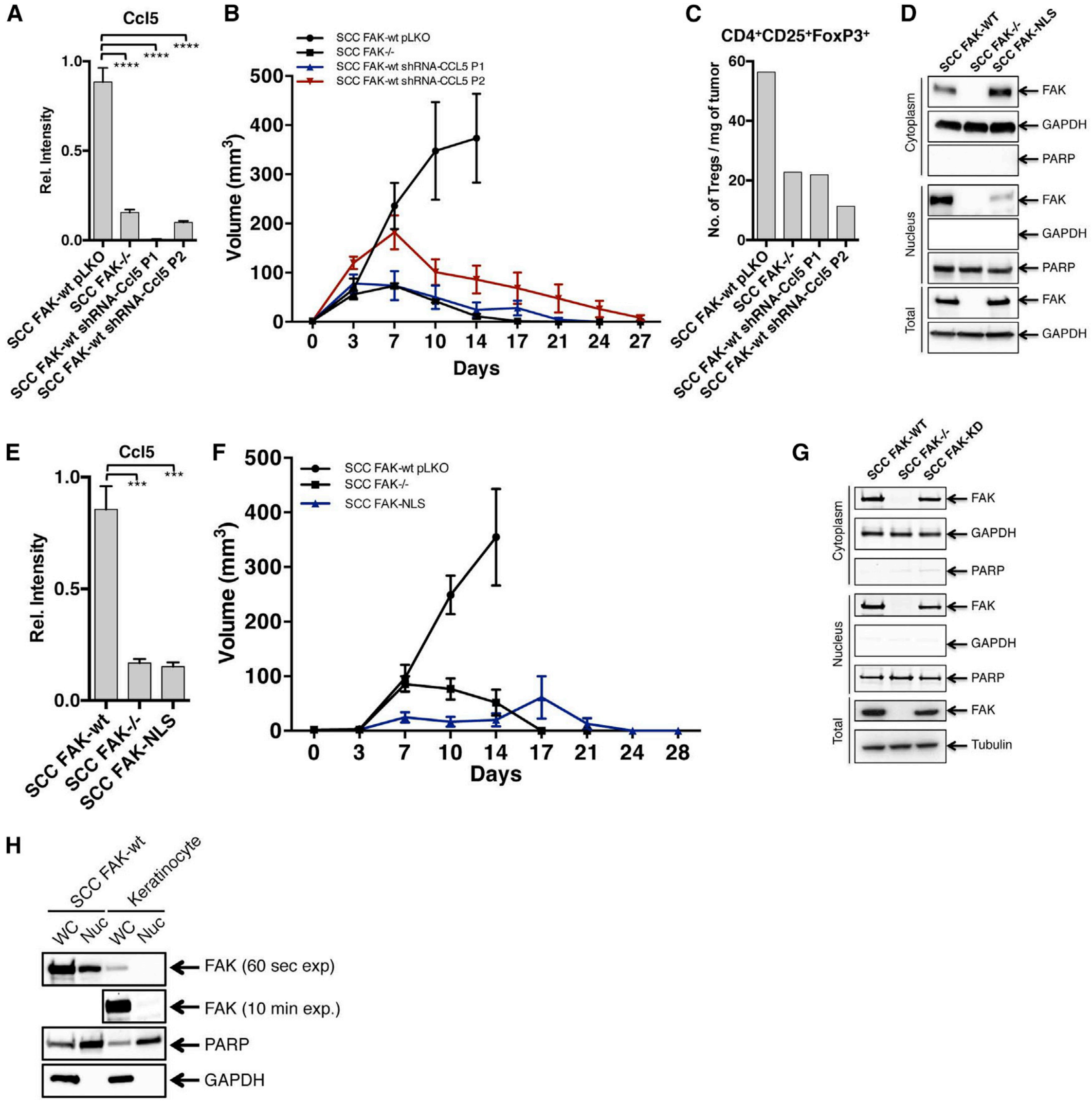

Figure 5. Nuclear FAK Regulates Transcription of Ccl5, which Is Required for Treg Recruitment and Tumor Growth

(A) qRT-PCR analysis of *Ccl5* gene expression knockdown in SCC FAK-WT cells stably expressing two independent shRNA constructs targeting Ccl5 (P1 and P2).

(B) SCC FAK-WT shRNA-Ccl5 tumor growth in FVB mice. n = 6 tumors.

(C) FACS quantitation of tumor-infiltrating Treg numbers from SCC FAK-WT shRNA-Ccl5 tumors. Data represent a single value from six pooled tumors.

(D) Western blotting of cytoplasmic, nuclear, and total protein fractions from SCC FAK-WT, SCC *FAK*$^{-/-}$, and SCC FAK-NLS cells.

(E) qRT-PCR analysis of *Ccl5* gene expression in SCC FAK-NLS cells.

(F) Tumor growth of SCC FAK-NLS cells in FVB mice.

(G) Western blotting of cytoplasmic, nuclear, and total protein fractions from SCC FAK-WT, SCC *FAK*$^{-/-}$, and SCC FAK-KD cells.

(H) Western blotting of whole-cell (WC) and nuclear (Nuc) protein fractions from SCC FAK-WT cells and primary skin keratinocytes. 60 s exposure time is shown for all samples; additional 10 min exposure time is shown for FAK in keratinocyte samples. GAPDH, cytoplasmic; PARP, nuclear.

***$p < 0.001$, ****$p < 0.0001$; Tukey-corrected one-way ANOVA. Data are represented as mean ± SEM unless stated.

other group that did not display such frank ulceration, we observed tumor regression by day 27 (Figure S5C, dashed red line). Analysis of Treg levels in SCC FAK-WT shRNA-TGFβ2 tumors at day 7 (regardless of initial growth characteristics) revealed that TGFβ2 knockdown was also associated with a reduction in CD4$^+$FoxP3$^+$CD25$^+$ Tregs (Figure S5D). Therefore, while the effects of reducing TGFβ2 expression are more complicated than for Ccl5, FAK-dependent TGFβ2 expression does contribute to elevated CD4$^+$FoxP3$^+$CD25$^+$ Tregs in SCC FAK-WT tumors; and in the subset of mice bearing tumors that were able to complete the study, TGFβ2 knockdown also caused tumor regression.

Our findings that FAK regulated the transcription of cytokines and chemokines (including Ccl5 and TGFβ2) that were associated with elevated intra-tumoral Tregs and tumor tolerance led us to consider a possible role for nuclear FAK in regulating the transcription of these genes. Based on previous reports (Lim et al., 2008), which identified putative NLSs within the FERM domain of FAK, we constructed an optimally nuclear targeting-impaired mutant FAK by replacing two arginines (positions 177 and 178) and four lysines (positions 190, 191, 216, and 218) with alanines (termed FAK-NLS). Western blotting of cytoplasmic and nuclear fractions confirmed that the FAK-NLS mutant was indeed defective in nuclear localization (Figure 5D). Subsequent (q)RT-PCR analysis of *Ccl5* and *Tgfb2* expression in SCC cells expressing only FAK-NLS revealed that FAK nuclear localization was required for transcription of these genes (Figures 5E and S5E, respectively). Thus, nuclear FAK drives the transcription of Ccl5 and TGFβ2, which are required for recruitment and expansion of immuno-suppressive Tregs into SCC tumors, altering the balance between CD8$^+$ T cells and Tregs in favor of tumor tolerance. In support of this, growth of SCC FAK-NLS tumor cells was similar to that of SCC $FAK^{-/-}$, with ultimate tumor regression (Figure 5F). This confirmed that it was nuclear FAK that afforded protection from the anti-tumor immune response. Western blotting of cytoplasmic and nuclear fractions from SCC FAK-KD showed that the kinase-deficient mutant was able to localize to the nucleus, so we conclude that the immune modulatory effects of FAK are dependent on FAK kinase activity in the nucleus (Figure 5G).

We next examined nuclear FAK levels in primary skin keratinocytes, the normal cellular counterparts of the SCC cells used here, and did not find detectable nuclear FAK (Figure 5H). Thus, abundant nuclear localization, and therefore the capacity to exert regulatory control over chemokine and cytokine expression, is likely a feature of oncogenic transformation in skin keratinocytes. This suggests that the nuclear functions of FAK that we have identified—namely, regulating transcription of chemokine/cytokine networks—may be associated with the cancerous state when FAK is highly expressed.

Nuclear FAK Interacts with a Network of Ccl5 Transcriptional Regulators

Having established an important role for the nuclear FAK-dependent transcription of Ccl5 in mediating recruitment and expansion of intra-tumoral Tregs, we wanted to determine how nuclear FAK could exert control over Ccl5 transcription. Using sucrose gradients, we fractionated the nuclei of SCC FAK-WT cells and demonstrated that nuclear FAK was present in the chromatin-containing fraction (Figure 6A). Transcriptional regulation of *Ccl5* is mediated predominantly through six short regulatory elements contained within a region of the *Ccl5* promoter spanning ~300 base pairs (Fessele et al., 2002). These regulatory elements contain binding sites for a number of TFs, including AP-1, C/EBP, IRF-1, NF-κB, and TATA box-binding protein (TBP), which is part of the transcription factor IID complex (TFIID). Using FAK immunoprecipitation and quantitative label-free mass spectrometry, we identified FAK binding partners in purified nuclear extracts and contextualized these by mapping onto a network of proteins associated with predicted Ccl5 TFs (constructed in silico; Figure 6B). This integrative approach identified a subset of Ccl5 TFs and regulators of these that interact with FAK in SCC cell nuclei (Figures 6C, S6 and Table S1). Interaction network analysis of this protein subset revealed nuclear FAK binding partners with roles in multiple transcriptional pathways, including regulators of AP-1, C/EBP, IRF-1/-7, NF-κB/Rel, and TFIID. Thus, we identified nuclear FAK binding partners that can interact, directly or indirectly, with five of the six main regulatory elements reported to control transcription of Ccl5 in multiple cell types (Fessele et al., 2002). Given that our interaction network was somewhat dominated by proteins associated with the TFIID pathway, including three TBP-associated factors (TAFs) (Figures 6C and S6), we used co-immunoprecipitation to confirm the interaction of nuclear FAK with one of these, TAF9, a core component of the TFIID complex (D'Alessio et al., 2009) (Figure 6D). Our data show that FAK binds to core components of the transcriptional machinery, many of which are known to be located on the promoter of genes undergoing active transcription and that are known or predicted to regulate Ccl5. Therefore, in SCC cells, nuclear FAK associates with chromatin and is physically linked to a network of TFs and their regulators known to modulate Ccl5 expression.

Small-Molecule FAK Kinase Inhibitor Promotes Immune-Mediated Tumor Clearance

Therapeutic targeting of FAK kinase activity using small-molecule inhibitors will inhibit FAK signaling not only in tumor cells, but also potentially in multiple host cell types. To complement expression of the FAK-KD mutant protein in the cancer cells and investigate whether a FAK inhibitor could induce immune-mediated regression of SCC tumors, we used the FAK/Pyk2 kinase inhibitor VS-4718 (Shapiro et al., 2014), which is currently in clinical development. Mice were treated with VS-4718 at 75 mg/kg for 24 hr prior to injection of 1 × 10^6 FAK-WT or $FAK^{-/-}$ SCC tumor cells and twice daily thereafter. This resulted in VS-4718-induced regression of SCC FAK-WT tumors by day 24 (Figure 7A). Following cessation of VS-4718 treatment, no tumor regrowth was observed (data not shown). SCC $FAK^{-/-}$ tumor growth and clearance was not greatly affected by VS-4718 treatment, suggesting that the anti-tumor effects of VS-4718 can be explained by FAK inhibition in tumor cells. Activity of VS-4718 was confirmed using an ELISA to measure FAK autophosphorylation on tyrosine-397 in tumor lysates from mice treated with 75 mg/kg VS-4718 (Figure S7). Regression of VS-4718-treated SCC tumors was not accompanied by loss of cell viability at day 7, as measured by FACS using a viability stain following tumor disaggregation (Figure 7B). There was a significant but small increase in leukocytes in VS-4718-treated SCC FAK-WT tumors (Figure 7C) and a significant increase in total CD4$^+$ T cells (Figures 7D and S2 and Table S2) and effector CD4$^+$CD44^{hi}CD62L^{low} T cells (Figures 7E and S2 and Table S2). A significant increase in CD8$^+$ T cells was also evident in SCC FAK-WT VS-4718-treated tumors (Figures 7F and S2 and Table S2), although there was no change in effector CD8$^+$CD44^{hi}CD62L^{low} T cells (Figures 7G and S2 and Table S2). Crucially, there was a significant reduction in CD4$^+$CD25$^+$FoxP3$^+$ Treg cells in VS-4718-treated SCC FAK-WT tumors, which was similar to that observed in vehicle and

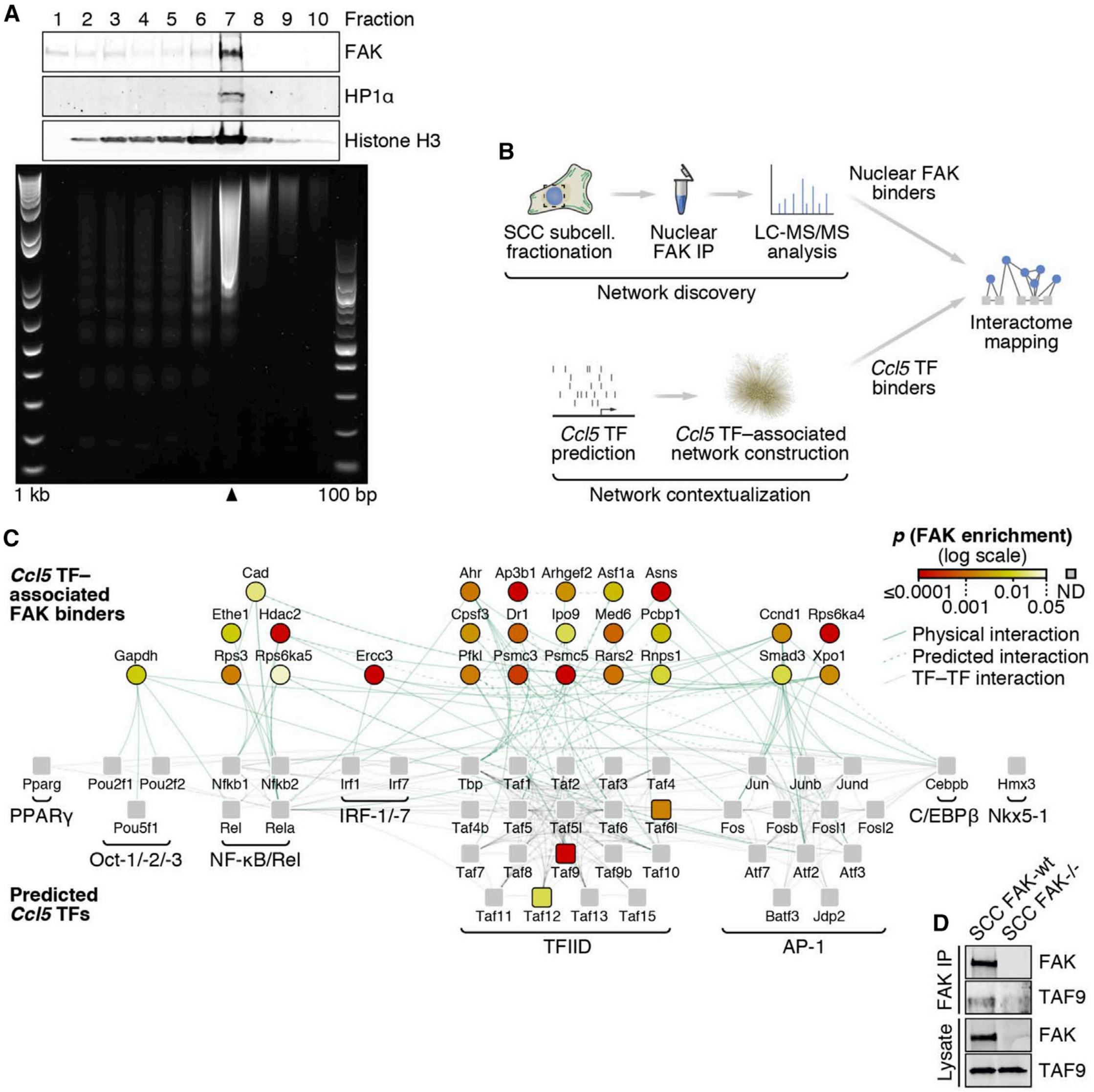

Figure 6. Nuclear FAK Interacts with Regulators of Ccl5 Transcription

(A) Sucrose fractionation of soluble chromatin prepared from SCC FAK-WT cell nuclei. Protein preparations recovered from each fraction were analyzed by western blotting (top). DNA recovered from each fraction was analyzed by agarose gel electrophoresis (bottom, 1 kilobase [kb] and 100 base pair [bp] ladders shown). Fraction 7 (black arrowhead) represents the chromatin-containing fraction.

(B) Schematic detailing the workflow used for proteomic analysis of the nuclear FAK interactome in the context of *Ccl5* transcription factors (TFs).

(C) Interaction network analysis of proteins that bind FAK in the nucleus of SCC cells. Predicted *Ccl5* TFs (squares, bottom) and respective TF binders (circles, top) enriched by at least 4-fold in nuclear FAK immunoprecipitations (SCC FAK-WT over SCC $FAK^{-/-}$ controls; $p < 0.05$) are shown (stringent network). *Ccl5* TFs not detected (ND) are shown as gray squares. TF complexes or groups are indicated; proteins are labeled with gene names for clarity. TF binders are aligned above TF groups with which there are the greatest number of reported interactions. For full network, see Figure S6; for protein interaction list, see Table S1.

(D) Isolation of the TFIID component TAF9 by FAK immunoprecipitation (IP) from SCC FAK-WT cell nuclear extracts.

VS-4718-treated SCC $FAK^{-/-}$ tumors (Figures 7H and S4 and Table S2).

Thus, VS-4718 promoted robust anti-tumor activity, with similar immune cell changes to that observed upon FAK deletion or expression of a kinase-deficient form of FAK. Furthermore, anti-tumor efficacy of VS-4718 was also dependent on $CD8^+$ T cells, and SCC FAK-WT tumors treated with VS-4718 on a $CD8^+$ T cell-depleted background exhibited a growth delay but did not undergo tumor regression (Figure 7I). We conclude that the FAK kinase inhibitor targets mechanisms of immune suppression and may therefore represent a form of effective "immuno-modulatory" therapy that reduces Tregs in the tumor environment. Importantly, the FAK kinase inhibitor does not affect the cytotoxic function of antigen-primed $CD8^+$ T cells. We also found that VS-4718 treatment that was initiated 5 days post-inoculation of 1×10^6 SCC FAK-WT cells, when these had

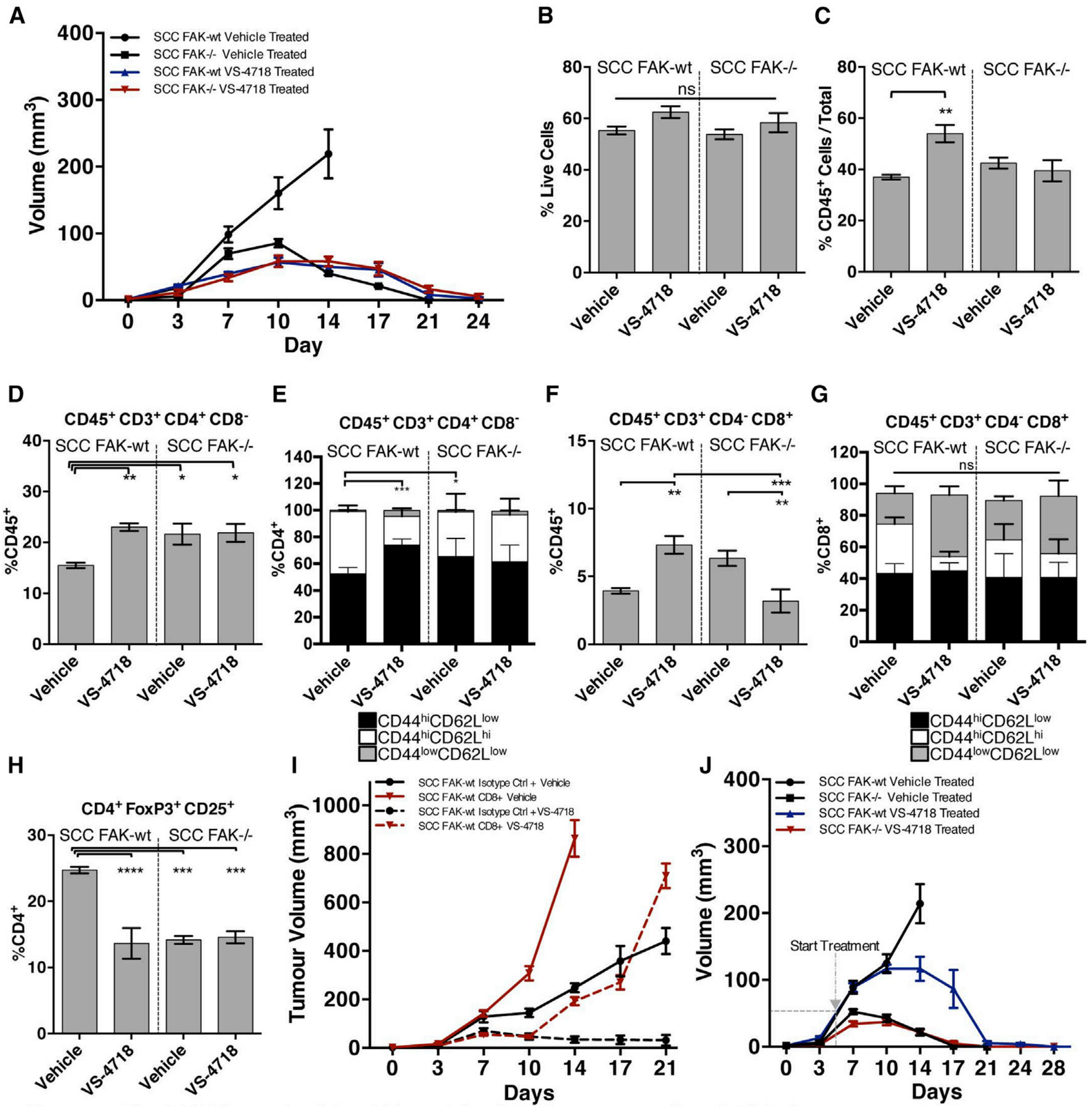

Figure 7. The FAK Kinase Inhibitor VS-4718 Leads to Immune-Mediated SCC Clearance

(A) SCC FAK-WT and SCC $FAK^{-/-}$ tumor growth in FVB mice treated with either vehicle or VS-4718. Treatment started 24 hr pre-tumor cell inoculation and continued for the duration of the experiment.

(B) FACS analysis of cell viability from disaggregated tumors treated with either vehicle or VS-4718.

(C) FACS analysis of vehicle- or VS-4718-treated tumor-infiltrating leukocytes expressed as a percentage of viable $CD45^+$ cells relative to the total number of single cells.

(D) FACS analysis of tumor-infiltrating $CD4^+$ T cells from vehicle- or VS-4718-treated tumors.

(E) FACS sub-categorization of tumor-infiltrating $CD4^+$ T cells into $CD45^+CD3^+CD4^+CD8^-CD44^{hi}CD62L^{low}$, $CD45^+CD3^+CD4^+CD8^-CD44^{hi}CD62L^{hi}$, and $CD45^+CD3^+CD4^+CD8^-CD44^{low}CD62L^{low}$ populations.

(F) FACS analysis of tumor-infiltrating $CD8^+$ T cells from vehicle- or VS-4718-treated tumors.

(G) FACS sub-categorization of tumor-infiltrating $CD8^+$ T cells into $CD45^+CD3^+CD4^-CD8^+CD44^{hi}CD62L^{low}$, $CD45^+CD3^+CD4^-CD8^+CD44^{hi}CD62L^{hi}$, and $CD45^+CD3^+CD4^-CD8^+CD44^{low}CD62L^{low}$ populations.

(H) FACS analysis of tumor-infiltrating $CD4^+CD25^+FoxP3^+$ Tregs expressed as a percentage of tumor-infiltrating $CD4^+$ T cells.

(I) SCC FAK-WT tumor growth in FVB mice treated with either vehicle or VS-4718 and either isotype control or CD8-depleting antibodies.

(J) SCC FAK-WT and SCC $FAK^{-/-}$ tumor growth in FVB mice treated with either vehicle or VS-4718. Treatment started 5 days post-tumor cell inoculation (gray dashed line) and continued for the duration of the experiment.

*$p < 0.05$, **$p < 0.01$, ***$p < 0.001$, ****$p < 0.0001$; ns, not significant; Tukey-corrected one-way ANOVA (E and G, $CD44^{hi}CD62L^{low}$ only). Data are represented as mean ± SEM; n = 6 tumors.

already formed palpable tumors of ~50 mm^3, led to complete tumor regression (Figure 7J).

DISCUSSION

We show that nuclear FAK in SCC cancer cells drives the transcription of chemokines and cytokines, including Ccl5 and TGFβ2, which promote the formation of an immuno-suppressive, pro-tumorigenic microenvironment. This is dependent on FAK kinase activity, and expression of a catalytically inactive mutant FAK protein (FAK-KD) or treatment with a small-molecule inhibitor causes tumor regression. This is effective even when tumors are already established, raising the exciting possibility that targeting of FAK kinase activity may have immune-mediated anti-tumor efficacy in patients. We established that nuclear FAK is associated with chromatin and interacts with a number of TFs and transcriptional regulators, including components of the TFIID complex, that are linked to regulation of Ccl5 expression. Our data imply that FAK interacts with core transcriptional machinery to influence gene transcription and promote tumor immune escape.

Historically, FAK has been recognized as an adhesion-related non-receptor protein tyrosine kinase that clusters at focal adhesion (FA) structures and regulates cancer-associated processes, including adhesion, migration, invasion, survival, and proliferation (reviewed in Frame et al., 2010). FAK was also found to translocate to the nucleus (Lim et al., 2008; Luo et al., 2009b), leading to the idea of nuclear functions for FAK within the nucleus. Our data show that, at least in cancer cells, FAK regulates inflammatory transcriptional programs associated with generation and maintenance of a pro-tumorigenic and immuno-suppressive microenvironment. FAK associates with chromatin, and in the context of Ccl5 expression, it interacts with a number of TFs, and regulators of TFs, that bind regulatory elements in the Ccl5 promoter (Fessele et al., 2002). Our data imply that FAK exists in complexes with a number of TAF proteins, including TAF9 and TAF12, key components of the core promoter complex TFIID that serves to initiate transcription by driving recruitment of chromatin remodeling complexes, coactivators, and RNA polymerase II to the promoter (D'Alessio et al., 2009). Therefore, FAK interacts with components of the core transcriptional machinery in order to drive transcription of chemokines and cytokines that contribute to recruitment of Tregs into the tumor environment, promoting immunological tolerance and permitting tumor growth.

Recently, nuclear accumulation of active FAK (phosphorylated on Tyr-397) within tumor cells of patients with colorectal cancer was reported to correlate with poor prognosis (Albasri et al., 2014), highlighting the need to understand the nature of FAK's role within the nucleus. Studies using endothelial cells, muscle cells, and fibroblasts have previously reported low steady-state levels of nuclear FAK that are substantially increased in response to cellular stress (Lim, 2013; Lim et al., 2008; Luo et al., 2009b). Our work implies that oncogenic stress is another route to inducing high levels of nuclear FAK and that this, in turn, can influence transcriptional programs, such as the chemokine and cytokine networks that control the tumor microenvironment.

A number of therapeutic strategies targeting components of the immuno-suppressive tumor microenvironment are currently being tested, with the aim of restoring anti-tumor immunity by releasing the break on CD8$^+$ T cell cytotoxic activity. In pre-clinical models of cancer, targeting Tregs (Ali et al., 2014; Bos et al., 2013) has shown anti-tumor efficacy, either alone or when used in combination with agents that enhance CD8$^+$ T cell activation. A clinical study combining agents targeting cytotoxic-T-lymphocyte-associated antigen 4 (CTLA-4), which is thought to influence Treg function (Peggs et al., 2009; Quezada et al., 2006; Simpson et al., 2013; Wing et al., 2008), and PD-1, which blocks signals that inhibit T cell function, has reported impressive responses in patients with advanced melanoma (Wolchok et al., 2013). However, this combination of checkpoint blockade antibodies elicits substantial side effects in >50% of patients, highlighting the need to find alternative combinations with improved tolerability. We have shown that targeting FAK kinase activity has the potential to modulate intra-tumoral Treg levels, resulting in robust CD8$^+$ T cell anti-tumor immunity, while others have reported previously that FAK kinase inhibitors block monocyte/macrophage and cancer-associated fibroblast recruitment into tumors by virtue of FAK's role in regulating their migration (Stokes et al., 2011). Taken together, these findings suggest that targeting the pleiotropic cellular functions of FAK may have a broad impact on the immuno-suppressive tumor microenvironment, differentiating these agents from many therapeutic approaches that target single immune cell populations.

Targeting a molecular pathway that is upregulated in cancer cells may provide tumor specificity and help to overcome some of the potential issues with severe autoimmunity when modulating immune cell populations. FAK inhibitors, such as VS-4718, are in clinical development. VS-4718 is currently in a phase I dose escalation clinical trial in patients with solid tumors (www.clinicaltrials.gov NCT01849744). Our findings provide good rationale for pre-clinical and clinical testing of FAK kinase inhibitors alongside agents that stimulate CD8$^+$ T cell activity, such as the checkpoint blockade therapies that target PD-1 and CTLA-4, which are both in clinical development (Pardoll, 2012).

EXPERIMENTAL PROCEDURES

Experiments involving animals were carried out in accordance with the UKCCCR guidelines by approved protocol (HO PL 60/4248). Brief experimental procedures are listed here. For details, please see the Supplemental Experimental Procedures.

Generation of FAK Nuclear Localization Mutant

Mutations were introduced into FAK-WT at R177A, R178A, K190A, K191A, K216A, and K218A using PCR-based site-directed mutagenesis.

Cell Lines

Isolation and generation of the FAK SCC cell model is described in Serrels et al. (2012). Keratinocyte cultures were prepared as detailed in McLean et al. (2004).

Western Blot Analysis

To prepare whole-cell lysates, cells were washed in cold PBS and lysed in RIPA buffer. Cytoplasmic and nuclear extracts were prepared as described in Lim et al. (2008). Lysates were resolved by gel electrophoresis, transferred to nitrocellulose, and probed with respective antibodies.

Subcutaneous Tumor Growth

Cells were injected into both flanks of either CD-1 nude mice or FVB mice and tumor growth measured twice-weekly. Animals were sacrificed when tumors reached maximum allowed size or when signs of ulceration were evident. For treatment with VS-4718, drug was prepared in 0.5% carboxymethyl cellulose + 0.1% Tween 80 and mice treated at 75 mg/kg BID by gavage. No signs of toxicity were observed.

Tumor Growth following Re-Challenge

SCC $FAK^{-/-}$ cells were injected into the left flank of FVB mice. Following tumor regression, mice were left for 7 days before being challenged with SCC FAK-WT or $FAK^{-/-}$ cells injected into the right flank. Tumor growth was measured twice-weekly. Control groups were injected into both flanks at day 28 using mice that had not been pre-challenged with SCC $FAK^{-/-}$ cells.

$CD4^+$, $CD8^+$, and $CD25^+$ T Cell Depletion

T cell depletion was achieved following IP injection of 150 μg of depleting antibody into female age-matched FVB mice for 3 consecutive days and was maintained by further IP injection at 3 day intervals until the study was terminated. SCC FAK-WT or $FAK^{-/-}$ cells were injected into both flanks 6 days after initial antibody treatment and tumor growth measured. The extent of T cell depletion was determined at the end of the study using FACS (Figure S1).

FACS Analysis of Immune Cell Populations

Tumors established following injection of SCC cells into both flanks of an FVB mouse were removed at day 7. Tumor tissue was processed to obtain single cell suspension for staining and subsequent FACS analysis (antibodies listed in Table S2).

Gene Expression Profiling

RNA was analyzed using the GeneChip Mouse Genome 430 2.0 Array. Normalized data for differentially expressed genes were median centered and clustered using Cluster 3.0 and Java TreeView. Functional enrichment analysis was performed using ToppGene.

Quantitative RT^2-PCR Array Analysis of Cytokine, Chemokine, and Chemokine Receptor Expression

RNA prepared from SCC cells was analyzed using the mouse cytokine and chemokine RT^2 Profiler PCR Array and that from isolated Tregs was analyzed using the mouse chemokine and receptor array. Relative gene expression ($2^{-\Delta Ct}$) values were log transformed, median centered, and subjected to hierarchical clustering as for microarray analysis. An interactome of chemokine ligands and receptors was constructed using the IUPHAR/BPS Guide to Pharmacology database and curated from the literature, onto which expression data for detected genes were mapped and visualized using Cytoscape. Expression of selected cytokine and chemokine genes was assessed by standard quantitative RT-PCR.

shRNA-Mediated TGFβ2 and Ccl5 Knockdown

Cells were subject to two rounds of lentiviral infection prior to selection with puromycin. shRNA constructs used were part of the pLKO lentiviral TRC library.

Preparation and Fractionation of Nuclei and Chromatin

Nuclei were prepared as described (Gilbert et al., 2003) but with a reduced concentration (0.05%) of NP-40 in nuclei buffer B. Soluble chromatin was prepared as described (Gilbert et al., 2004) and fractionated on a sucrose step gradient to separate soluble and chromatin-associated nuclear proteins. DNA was recovered from fractions and subjected to agarose gel electrophoresis. Protein was purified using TCA precipitation. Samples were analyzed by SDS-PAGE and blotted using anti FAK, HP1α, and histone H3 antibodies.

Proteomic Analysis of Nuclear FAK Protein Complexes

FAK nuclear protein complexes were subjected to on-bead proteolytic digestion, desalting, and liquid chromatography-tandem mass spectrometry, as described (Turriziani et al., 2014). For interaction network analysis, Ccl5 transcription factors were extracted from the DECODE database and used to seed a network of 1,000 transcription factor-related proteins using the GeneMANIA plugin in Cytoscape. Proteins specifically isolated in nuclear FAK protein complexes were mapped onto the interactome, and those with physical or predicted direct or indirect interactions with Ccl5 transcription factors were analyzed using the NetworkAnalyzer plugin in Cytoscape.

CD8 T Cell Fluorescent Immunohistochemistry

Tumors were removed 7 days post-implantation and frozen by submersing in liquid nitrogen. Tumor sections were cut, processed and stained. They were imaged using an Olympus FV1000 confocal microscope.

ACCESSION NUMBERS

The microarray data discussed in this manuscript has been deposited in NCBI's Gene Expression Omnibus and is accessible through GEO series accession number GEO: GSE71662.

SUPPLEMENTAL INFORMATION

Supplemental Information includes Supplemental Experimental Procedures, seven figures, and two tables and can be found with this article online at http://dx.doi.org/10.1016/j.cell.2015.09.001.

AUTHOR CONTRIBUTIONS

A.S. and M.C.F. devised and oversaw the project. A.S., T.L., B.S., A.B., S.M.A., R.J.B.N., and M.C.F. designed the experiments with contributions from E.M., J.A.P., V.G.B., and N.G. A.S., T.L., B.S., A.B., R.C.M., and A.v.K. performed experiments with contributions from L.G.-C., M.C., M.M., and J.E.R. A.S., T.L., B.S., A.B., R.C.M., A.v.K., and A.H.S. analyzed the data. A.B. and A.H.S. performed bioinformatic analysis. A.S. and M.C.F. wrote the manuscript with contributions from T.L., B.S., and A.B.; all authors commented on and approved the final version. We consider that A.S. and T.L. made equal contributions and that B.S. and A.B. made equal contributions.

ACKNOWLEDGMENTS

This work was supported by Cancer Research UK (Grant no. C157/A15703 to M.C.F.), European Research Council (Grant no. 29440 Cancer Innovation to M.C.F.) and Medical Research Council (Grant no. G1100084 to S.M.A.). We thank Frederic Li Mow Chee for help with transcriptomic analysis, Amaya García-Muñoz for help with mass spectrometry, Elisabeth Freyer for help with FACS, and Arkadiusz Welman for help with manuscript preparation. J.E.R. and J.A.P. are employees of Verastem Inc.

Received: March 6, 2015
Revised: July 17, 2015
Accepted: August 27, 2015
Published: September 24, 2015

REFERENCES

Albasri, A., Fadhil, W., Scholefield, J.H., Durrant, L.G., and Ilyas, M. (2014). Nuclear expression of phosphorylated focal adhesion kinase is associated with poor prognosis in human colorectal cancer. Anticancer Res. *34*, 3969–3974.

Ali, K., Soond, D.R., Piñeiro, R., Hagemann, T., Pearce, W., Lim, E.L., Bouabe, H., Scudamore, C.L., Hancox, T., Maecker, H., et al. (2014). Inactivation of PI(3) K p110δ breaks regulatory T-cell-mediated immune tolerance to cancer. Nature *510*, 407–411.

Ashton, G.H., Morton, J.P., Myant, K., Phesse, T.J., Ridgway, R.A., Marsh, V., Wilkins, J.A., Athineos, D., Muncan, V., Kemp, R., et al. (2010). Focal adhesion kinase is required for intestinal regeneration and tumorigenesis downstream of Wnt/c-Myc signaling. Dev. Cell *19*, 259–269.

Beyer, M., and Schultze, J.L. (2006). Regulatory T cells in cancer. Blood *108*, 804–811.

Biragyn, A., and Longo, D.L. (2012). Neoplastic "Black Ops": cancer's subversive tactics in overcoming host defenses. Semin. Cancer Biol. *22*, 50–59.

Bos, P.D., Plitas, G., Rudra, D., Lee, S.Y., and Rudensky, A.Y. (2013). Transient regulatory T cell ablation deters oncogene-driven breast cancer and enhances radiotherapy. J. Exp. Med. *210*, 2435–2466.

Curiel, T.J., Coukos, G., Zou, L., Alvarez, X., Cheng, P., Mottram, P., Evdemon-Hogan, M., Conejo-Garcia, J.R., Zhang, L., Burow, M., et al. (2004). Specific recruitment of regulatory T cells in ovarian carcinoma fosters immune privilege and predicts reduced survival. Nat. Med. *10*, 942–949.

D'Alessio, J.A., Wright, K.J., and Tjian, R. (2009). Shifting players and paradigms in cell-specific transcription. Mol. Cell *36*, 924–931.

Darrasse-Jèze, G., and Podsypanina, K. (2013). How numbers, nature, and immune status of foxp3(+) regulatory T-cells shape the early immunological events in tumor development. Front. Immunol. *4*, 292.

Fessele, S., Maier, H., Zischek, C., Nelson, P.J., and Werner, T. (2002). Regulatory context is a crucial part of gene function. Trends Genet. *18*, 60–63.

Fourcade, J., Sun, Z., Benallaoua, M., Guillaume, P., Luescher, I.F., Sander, C., Kirkwood, J.M., Kuchroo, V., and Zarour, H.M. (2010). Upregulation of Tim-3 and PD-1 expression is associated with tumor antigen-specific CD8+ T cell dysfunction in melanoma patients. J. Exp. Med. *207*, 2175–2186.

Frame, M.C., Patel, H., Serrels, B., Lietha, D., and Eck, M.J. (2010). The FERM domain: organizing the structure and function of FAK. Nat. Rev. Mol. Cell Biol. *11*, 802–814.

Gilbert, N., Boyle, S., Sutherland, H., de Las Heras, J., Allan, J., Jenuwein, T., and Bickmore, W.A. (2003). Formation of facultative heterochromatin in the absence of HP1. EMBO J. *22*, 5540–5550.

Gilbert, N., Boyle, S., Fiegler, H., Woodfine, K., Carter, N.P., and Bickmore, W.A. (2004). Chromatin architecture of the human genome: gene-rich domains are enriched in open chromatin fibers. Cell *118*, 555–566.

Goldstein, J.D., Pérol, L., Zaragoza, B., Baeyens, A., Marodon, G., and Piaggio, E. (2013). Role of cytokines in thymus- versus peripherally derived-regulatory T cell differentiation and function. Front. Immunol. *4*, 155.

Kono, K., Kawaida, H., Takahashi, A., Sugai, H., Mimura, K., Miyagawa, N., Omata, H., and Fujii, H. (2006). CD4(+)CD25high regulatory T cells increase with tumor stage in patients with gastric and esophageal cancers. Cancer Immunol. Immunother. *55*, 1064–1071.

Lahlou, H., Sanguin-Gendreau, V., Zuo, D., Cardiff, R.D., McLean, G.W., Frame, M.C., and Muller, W.J. (2007). Mammary epithelial-specific disruption of the focal adhesion kinase blocks mammary tumor progression. Proc. Natl. Acad. Sci. USA *104*, 20302–20307.

Lim, S.T. (2013). Nuclear FAK: a new mode of gene regulation from cellular adhesions. Mol. Cells *36*, 1–6.

Lim, S.T., Chen, X.L., Lim, Y., Hanson, D.A., Vo, T.T., Howerton, K., Larocque, N., Fisher, S.J., Schlaepfer, D.D., and Ilic, D. (2008). Nuclear FAK promotes cell proliferation and survival through FERM-enhanced p53 degradation. Mol. Cell *29*, 9–22.

Luo, M., Fan, H., Nagy, T., Wei, H., Wang, C., Liu, S., Wicha, M.S., and Guan, J.L. (2009a). Mammary epithelial-specific ablation of the focal adhesion kinase suppresses mammary tumorigenesis by affecting mammary cancer stem/progenitor cells. Cancer Res. *69*, 466–474.

Luo, S.W., Zhang, C., Zhang, B., Kim, C.H., Qiu, Y.Z., Du, Q.S., Mei, L., and Xiong, W.C. (2009b). Regulation of heterochromatin remodelling and myogenin expression during muscle differentiation by FAK interaction with MBD2. EMBO J. *28*, 2568–2582.

Marigo, I., Dolcetti, L., Serafini, P., Zanovello, P., and Bronte, V. (2008). Tumor-induced tolerance and immune suppression by myeloid derived suppressor cells. Immunol. Rev. *222*, 162–179.

Matsuzaki, J., Gnjatic, S., Mhawech-Fauceglia, P., Beck, A., Miller, A., Tsuji, T., Eppolito, C., Qian, F., Lele, S., Shrikant, P., et al. (2010). Tumor-infiltrating NY-ESO-1-specific CD8+ T cells are negatively regulated by LAG-3 and PD-1 in human ovarian cancer. Proc. Natl. Acad. Sci. USA *107*, 7875–7880.

McLean, G.W., Komiyama, N.H., Serrels, B., Asano, H., Reynolds, L., Conti, F., Hodivala-Dilke, K., Metzger, D., Chambon, P., Grant, S.G., and Frame, M.C. (2004). Specific deletion of focal adhesion kinase suppresses tumor formation and blocks malignant progression. Genes Dev. *18*, 2998–3003.

McLean, G.W., Carragher, N.O., Avizienyte, E., Evans, J., Brunton, V.G., and Frame, M.C. (2005). The role of focal-adhesion kinase in cancer - a new therapeutic opportunity. Nat. Rev. Cancer *5*, 505–515.

Ondondo, B., Jones, E., Godkin, A., and Gallimore, A. (2013). Home sweet home: the tumor microenvironment as a haven for regulatory T cells. Front. Immunol. *4*, 197.

Onizuka, S., Tawara, I., Shimizu, J., Sakaguchi, S., Fujita, T., and Nakayama, E. (1999). Tumor rejection by in vivo administration of anti-CD25 (interleukin-2 receptor alpha) monoclonal antibody. Cancer Res. *59*, 3128–3133.

Pardoll, D.M. (2012). The blockade of immune checkpoints in cancer immunotherapy. Nat. Rev. Cancer *12*, 252–264.

Peggs, K.S., Quezada, S.A., Chambers, C.A., Korman, A.J., and Allison, J.P. (2009). Blockade of CTLA-4 on both effector and regulatory T cell compartments contributes to the antitumor activity of anti-CTLA-4 antibodies. J. Exp. Med. *206*, 1717–1725.

Provenzano, P.P., Inman, D.R., Eliceiri, K.W., Beggs, H.E., and Keely, P.J. (2008). Mammary epithelial-specific disruption of focal adhesion kinase retards tumor formation and metastasis in a transgenic mouse model of human breast cancer. Am. J. Pathol. *173*, 1551–1565.

Pylayeva, Y., Gillen, K.M., Gerald, W., Beggs, H.E., Reichardt, L.F., and Giancotti, F.G. (2009). Ras- and PI3K-dependent breast tumorigenesis in mice and humans requires focal adhesion kinase signaling. J. Clin. Invest. *119*, 252–266.

Quezada, S.A., Peggs, K.S., Curran, M.A., and Allison, J.P. (2006). CTLA4 blockade and GM-CSF combination immunotherapy alters the intratumor balance of effector and regulatory T cells. J. Clin. Invest. *116*, 1935–1945.

Roberts, W.G., Ung, E., Whalen, P., Cooper, B., Hulford, C., Autry, C., Richter, D., Emerson, E., Lin, J., Kath, J., et al. (2008). Antitumor activity and pharmacology of a selective focal adhesion kinase inhibitor, PF-562,271. Cancer Res. *68*, 1935–1944.

Sakuishi, K., Apetoh, L., Sullivan, J.M., Blazar, B.R., Kuchroo, V.K., and Anderson, A.C. (2010). Targeting Tim-3 and PD-1 pathways to reverse T cell exhaustion and restore anti-tumor immunity. J. Exp. Med. *207*, 2187–2194.

Sakuishi, K., Ngiow, S.F., Sullivan, J.M., Teng, M.W., Kuchroo, V.K., Smyth, M.J., and Anderson, A.C. (2013). TIM3(+)FOXP3(+) regulatory T cells are tissue-specific promoters of T-cell dysfunction in cancer. OncoImmunology *2*, e23849.

Sasada, T., Kimura, M., Yoshida, Y., Kanai, M., and Takabayashi, A. (2003). CD4+CD25+ regulatory T cells in patients with gastrointestinal malignancies: possible involvement of regulatory T cells in disease progression. Cancer *98*, 1089–1099.

Sato, E., Olson, S.H., Ahn, J., Bundy, B., Nishikawa, H., Qian, F., Jungbluth, A.A., Frosina, D., Gnjatic, S., Ambrosone, C., et al. (2005). Intraepithelial CD8+ tumor-infiltrating lymphocytes and a high CD8+/regulatory T cell ratio are associated with favorable prognosis in ovarian cancer. Proc. Natl. Acad. Sci. USA *102*, 18538–18543.

Serrels, A., McLeod, K., Canel, M., Kinnaird, A., Graham, K., Frame, M.C., and Brunton, V.G. (2012). The role of focal adhesion kinase catalytic activity on the proliferation and migration of squamous cell carcinoma cells. Int. J. Cancer *131*, 287–297.

Shah, W., Yan, X., Jing, L., Zhou, Y., Chen, H., and Wang, Y. (2011). A reversed CD4/CD8 ratio of tumor-infiltrating lymphocytes and a high percentage of CD4(+)FOXP3(+) regulatory T cells are significantly associated with clinical outcome in squamous cell carcinoma of the cervix. Cell. Mol. Immunol. *8*, 59–66.

Shapiro, I.M., Kolev, V.N., Vidal, C.M., Kadariya, Y., Ring, J.E., Wright, Q., Weaver, D.T., Menges, C., Padval, M., McClatchey, A.I., et al. (2014). Merlin deficiency predicts FAK inhibitor sensitivity: a synthetic lethal relationship. Sci. Transl. Med. *6*, 237ra68.

Shimizu, J., Yamazaki, S., and Sakaguchi, S. (1999). Induction of tumor immunity by removing CD25+CD4+ T cells: a common basis between tumor immunity and autoimmunity. J. Immunol. *163*, 5211–5218.

Simpson, T.R., Li, F., Montalvo-Ortiz, W., Sepulveda, M.A., Bergerhoff, K., Arce, F., Roddie, C., Henry, J.Y., Yagita, H., Wolchok, J.D., et al. (2013). Fc-dependent depletion of tumor-infiltrating regulatory T cells co-defines the efficacy of anti-CTLA-4 therapy against melanoma. J. Exp. Med. *210*, 1695–1710.

Slack-Davis, J.K., Hershey, E.D., Theodorescu, D., Frierson, H.F., and Parsons, J.T. (2009). Differential requirement for focal adhesion kinase signaling in cancer progression in the transgenic adenocarcinoma of mouse prostate model. Mol. Cancer Ther. *8*, 2470–2477.

Stokes, J.B., Adair, S.J., Slack-Davis, J.K., Walters, D.M., Tilghman, R.W., Hershey, E.D., Lowrey, B., Thomas, K.S., Bouton, A.H., Hwang, R.F., et al. (2011). Inhibition of focal adhesion kinase by PF-562,271 inhibits the growth and metastasis of pancreatic cancer concomitant with altering the tumor microenvironment. Mol. Cancer Ther. *10*, 2135–2145.

Tan, M.C., Goedegebuure, P.S., Belt, B.A., Flaherty, B., Sankpal, N., Gillanders, W.E., Eberlein, T.J., Hsieh, C.S., and Linehan, D.C. (2009). Disruption of CCR5-dependent homing of regulatory T cells inhibits tumor growth in a murine model of pancreatic cancer. J. Immunol. *182*, 1746–1755.

Thornton, A.M., Korty, P.E., Tran, D.Q., Wohlfert, E.A., Murray, P.E., Belkaid, Y., and Shevach, E.M. (2010). Expression of Helios, an Ikaros transcription factor family member, differentiates thymic-derived from peripherally induced Foxp3+ T regulatory cells. J. Immunol. *184*, 3433–3441.

Turriziani, B., Garcia-Munoz, A., Pilkington, R., Raso, C., Kolch, W., and von Kriegsheim, A. (2014). On-beads digestion in conjunction with data-dependent mass spectrometry: a shortcut to quantitative and dynamic interaction proteomics. Biology (Basel) *3*, 320–332.

Wherry, E.J. (2011). T cell exhaustion. Nat. Immunol. *12*, 492–499.

Wing, K., Onishi, Y., Prieto-Martin, P., Yamaguchi, T., Miyara, M., Fehervari, Z., Nomura, T., and Sakaguchi, S. (2008). CTLA-4 control over Foxp3+ regulatory T cell function. Science *322*, 271–275.

Wolchok, J.D., Kluger, H., Callahan, M.K., Postow, M.A., Rizvi, N.A., Lesokhin, A.M., Segal, N.H., Ariyan, C.E., Gordon, R.A., Reed, K., et al. (2013). Nivolumab plus ipilimumab in advanced melanoma. N. Engl. J. Med. *369*, 122–133.

Cell

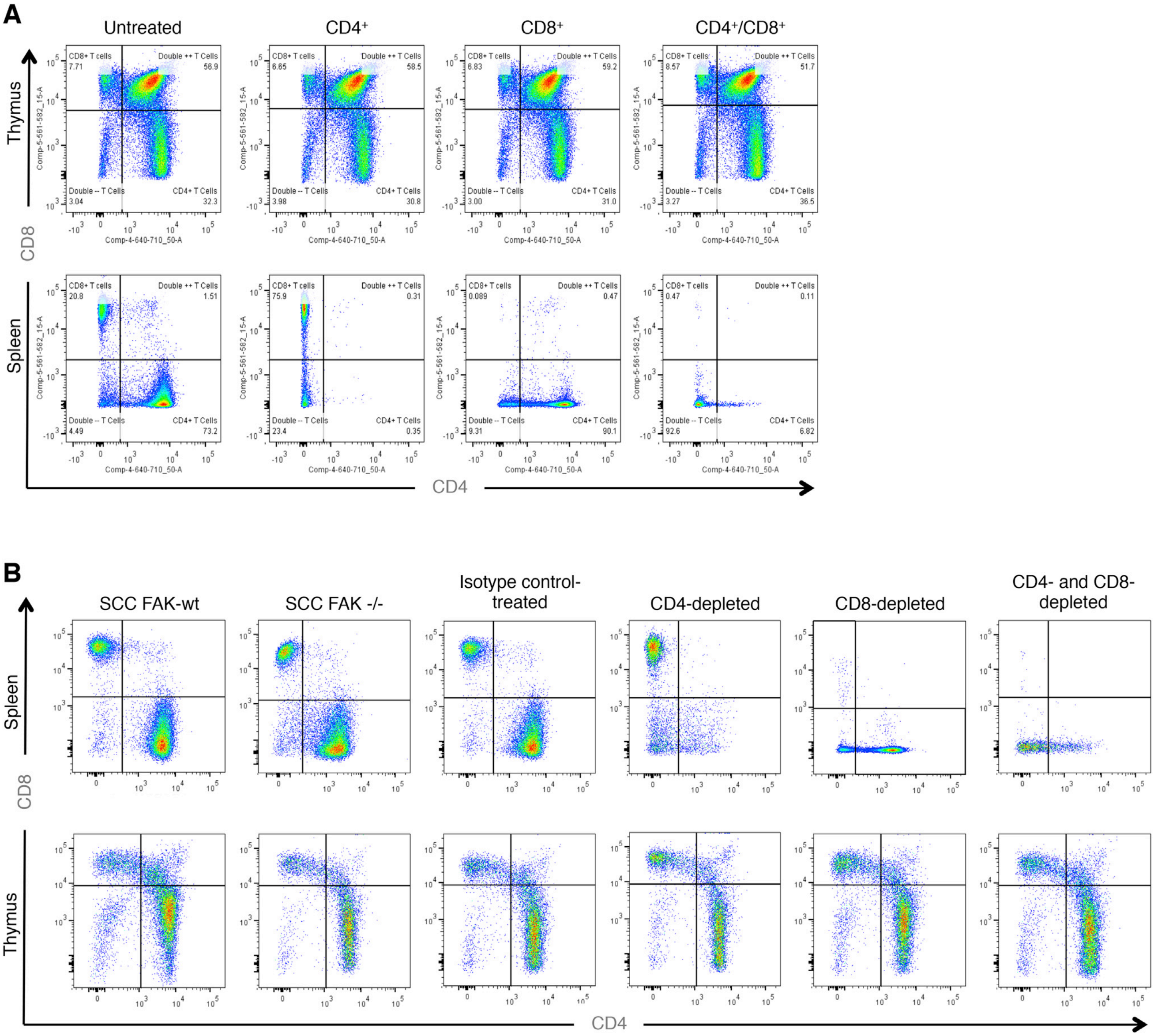

Figure S1. T Cell FACS Analysis Post Antibody-Mediated T Cell Depletion, Related to Figure 1

(A) FACS analysis of spleen and thymus tissue from non-tumor-bearing animals 6 days after commencing antibody treatment. (B) FACS analysis of T cell populations from spleen and thymus tissue from tumor-bearing animals at the end of T cell depletion studies in Figures 1C and 1D.

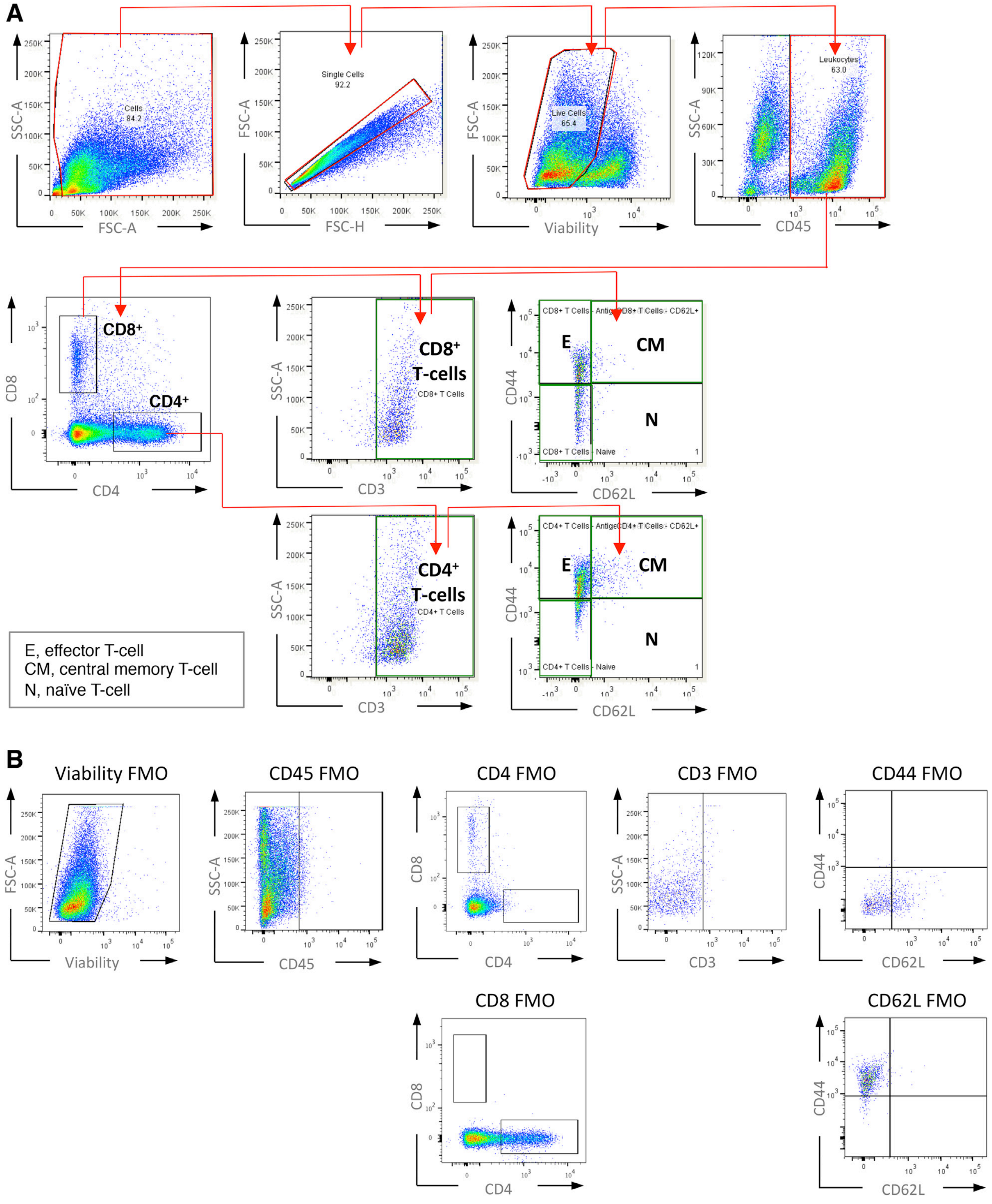

Figure S2. T Cell FACS Gating Strategy, Related to Figures 2 and 7

(A) FACS gating strategy applied for identification of T cell sub-populations. E = effector, CM = central memory, and N = naive. (B) FMO (full antibody set minus one) control samples used to determine correct gating for T cell sub-population identification.

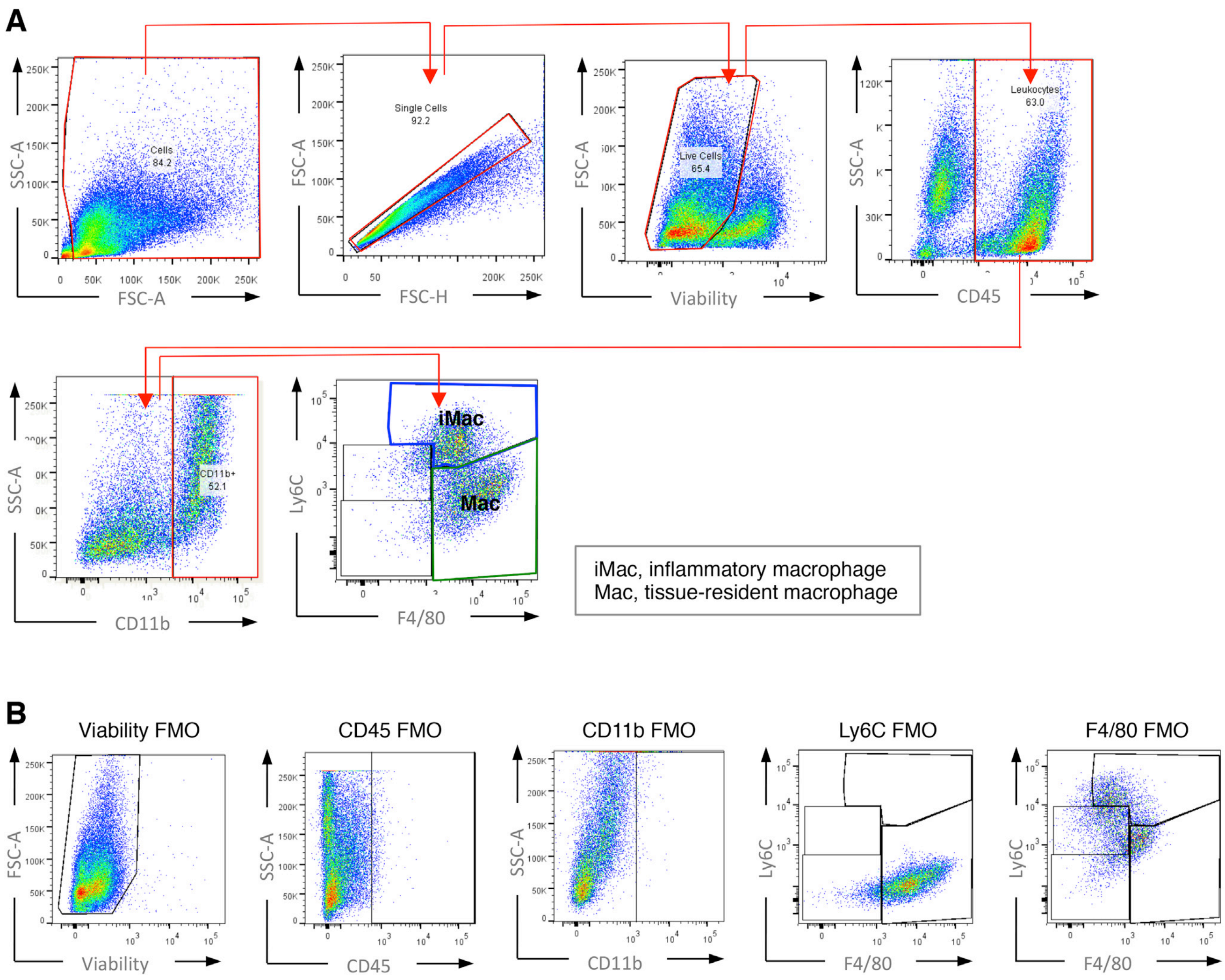

Figure S3. Macrophage FACS Gating Strategy, Related to Figure 3

(A) FACS gating strategy applied for identification of macrophage sub-populations. (B) FMO control samples used to determine correct gating for macrophage sub-population identification.

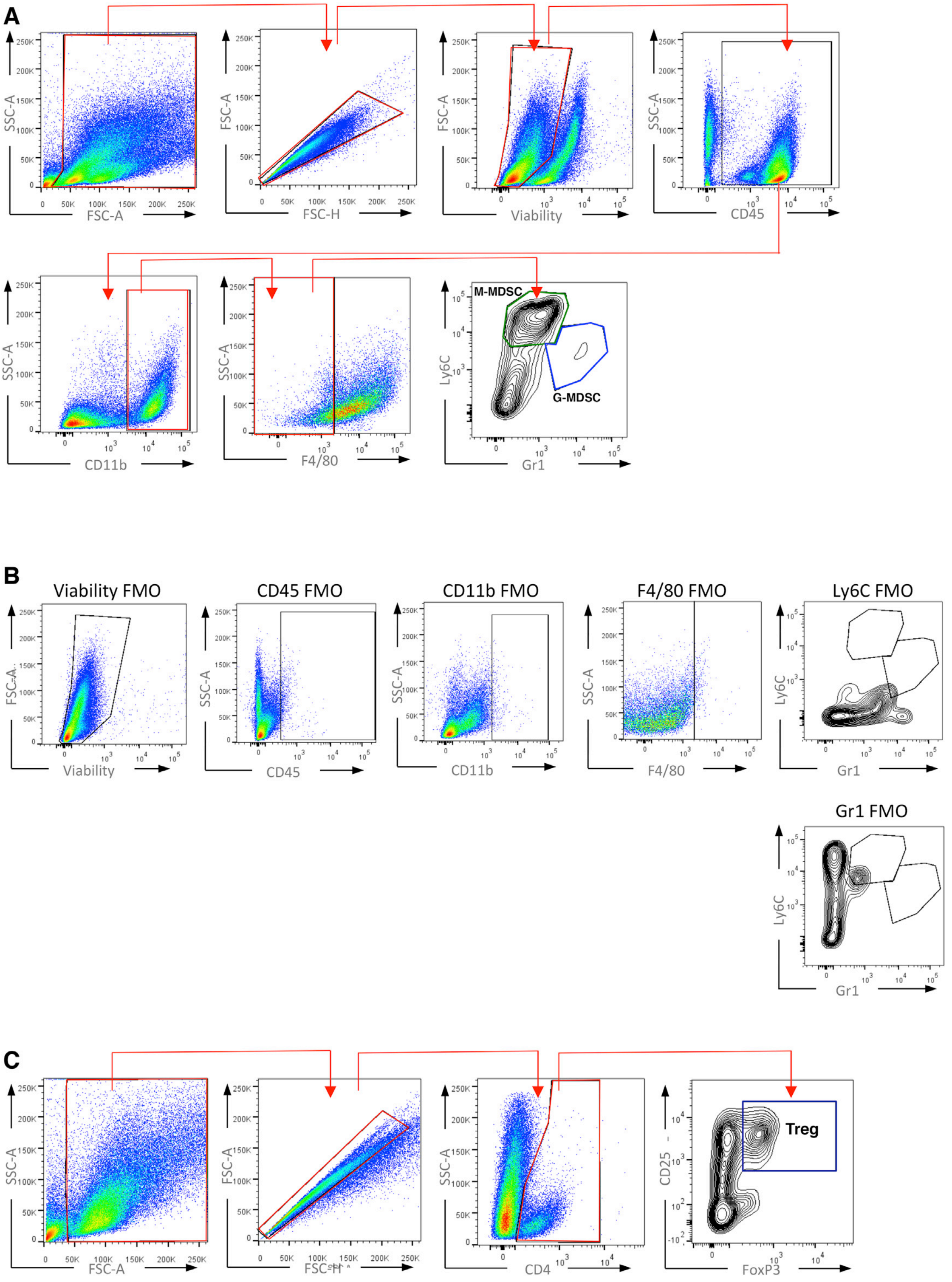

Figure S4. MDSC and Treg FACS Gating Strategy, Related to Figures 3 and 7

(A) FACS gating strategy applied for identification of MDSC sub-populations. M-MDSC – Monocytic Myeloid Derived Suppressor Cell; G-MDSC – Granulocytic Myeloid Derived Suppressor Cell. (B) FMO control samples used to determine correct gating for MDSC sub-population identification. (C) FACS gating strategy applied for identification of regulatory T cells (Treg).

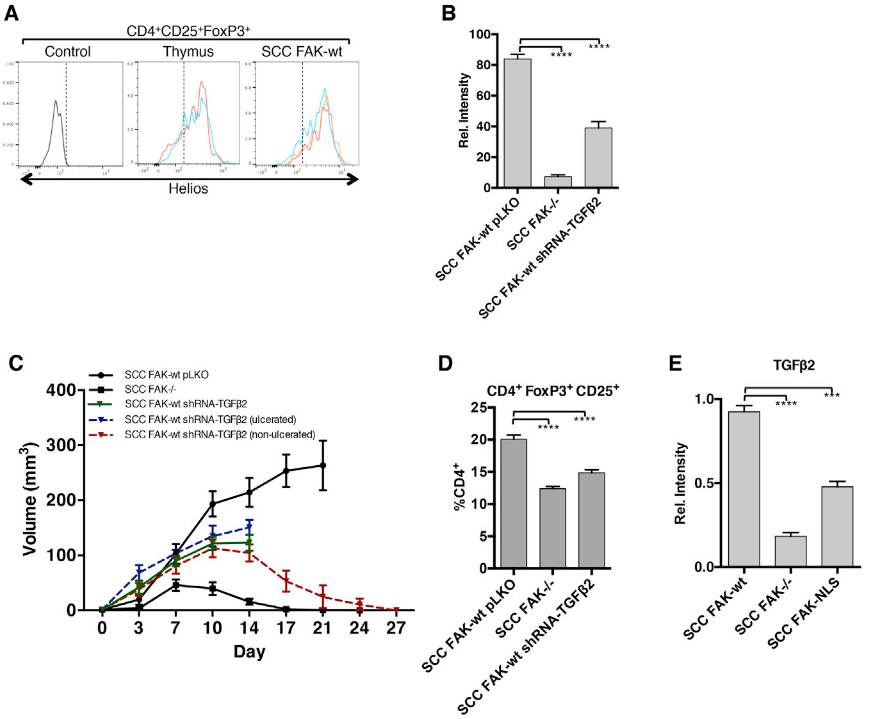

Figure S5. Tregs Infiltrating SCC FAK-WT Tumors Express the Thymic Marker Helios; Nuclear FAK Regulates Transcription of TGFβ2, which Contributes to Treg Expansion and Tumor Growth, Related to Figures 3 and 5

(A) FACS analysis of Helios expression in $CD4^+CD25^+FOXP3^+$ SCC FAK-WT tumor-infiltrating Tregs and Tregs isolated from the thymus of tumor-bearing mice. Control represents background signal from a sample stained with CD4, CD25, and FoxP3 conjugated antibodies but not Helios. Representative replicates are shown in different colors for thymus and SCC FAK-WT samples. (B) qRT-PCR analysis of *Tgfb2* gene expression knockdown in SCC cells. ****$p < 0.0001$ (Tukey-corrected one-way ANOVA). (C) SCC FAK-WT shRNA-TGFβ2 tumor growth in FVB mice. Blue dashed line indicates growth of SCC FAK-WT shRNA-TGFβ2 tumors that had to be sacrificed due to ulceration at day 14. Red dashed line indicates growth of SCC FAK-WT shRNA-TGFβ2 tumors that showed no ulceration. Green solid line indicates the mean growth of all SCC FAK-WT shRNA-TGFβ2 tumors up until cohort numbers were reduced due to ulceration. n = 6 tumors / group. (D) FACS analysis of SCC FAK-WT shRNA-TGFβ2 tumor infiltrating Tregs. ****$p < 0.0001$ (Tukey-corrected one-way ANOVA) (E) qRT-PCR analysis of *Tgfb2* gene expression in SCC FAK-NLS mutant cells. ***$p < 0.001$, ****$p < 0.0001$ (Tukey-corrected one-way ANOVA). Data are represented as mean ± SEM.

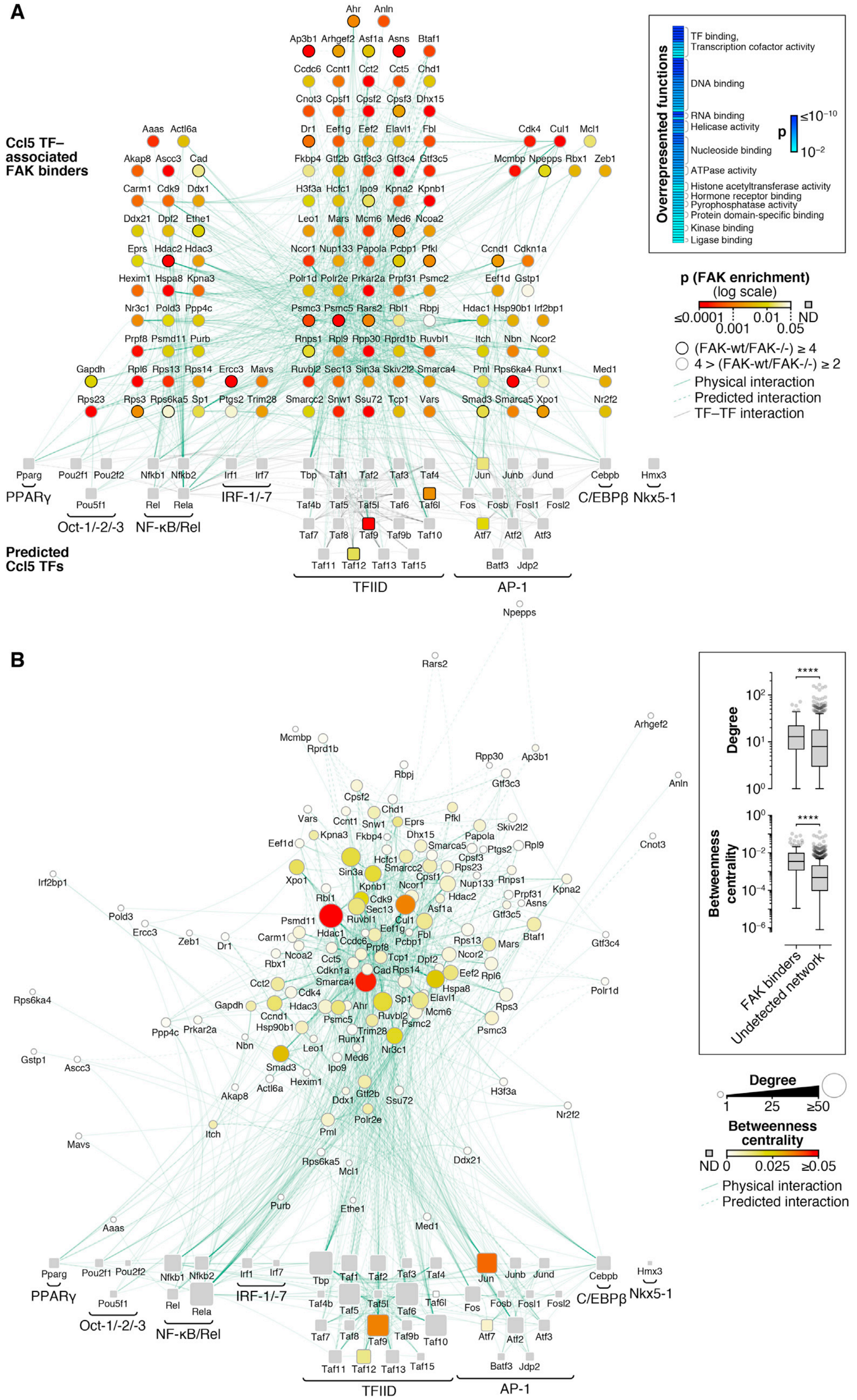

(legend on next page)

Figure S6. Nuclear FAK Interactome in the Context of Ccl5 Transcription Factors, Related to Figure 6 and Table S1

(A) Interaction network analysis of proteins that bind FAK in the nucleus of SCC cells. Predicted Ccl5 transcription factors (TFs) (squares; bottom) and respective TF binders (circles; top) enriched by at least two-fold in nuclear FAK immunoprecipitations (SCC FAK-WT over SCC *FAK*$^{-/-}$ controls; $p < 0.05$) are shown. Ccl5 TFs not detected (ND) are shown as gray squares. TF complexes or groups are indicated; proteins are labeled with gene names for clarity. TF binders are aligned above TF groups with which there are the greatest number of reported interactions. Overrepresented molecular functions determined by functional enrichment analysis are displayed as a heat map (log_{10}-transformed color scale) (inset). Displayed terms satisfy $p < 0.01$ (Benjamini–Hochberg-corrected hypergeometric test) with > 5 proteins assigned per term. (B) Topological analysis of Ccl5 TF–associated proteins identified in the nuclear FAK interactome. Ccl5 TF binders were clustered using the yFiles Organic algorithm implemented in Cytoscape. Topological parameters were computed using NetworkAnalyzer, excluding self-interactions. Protein node size is proportional to the number of interaction partners in the network (degree); node color indicates betweenness centrality (normalized number of shortest paths between proteins; a measure of the control a protein exerts over the interactions of other proteins in the network). Box-and-whisker plots (inset) show the distributions of degree and betweenness centrality for Ccl5 TF–associated proteins that bind nuclear FAK compared to those that were not enriched in nuclear FAK immunoprecipitations, indicating that FAK binders tend to have more interactions and be more central in the interaction network than undetected Ccl5 TF–associated proteins. Plots display the median (line), interquartile range (box) and 1.5 × interquartile range (whiskers) (n = 169 and 761 Ccl5 TF–associated proteins detected and not detected, respectively, with degree ≥ 1 based on physical or predicted interactions). ****$p < 0.0001$ (two-tailed Mann–Whitney test).

See also Table S1.

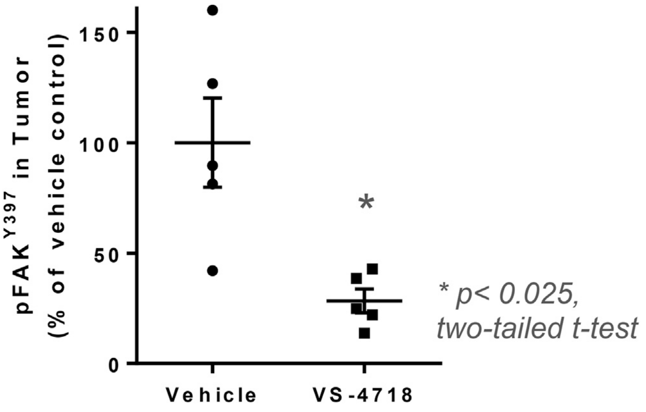

Figure S7. Analysis of FAK pY397 Phosphorylation in Tumors following Treatment with VS-4718, Related to Figure 7

Phosphorylation of FAK on Y397 was measured in protein lysates isolated from tumors following treatment with VS-4718 using ELISA. Tumors were removed within 30 min of treatment. n = 5. Data are represented as mean ± SEM.

Cell

Supplemental Information

Nuclear FAK Controls Chemokine Transcription, Tregs, and Evasion of Anti-tumor Immunity

Alan Serrels, Tom Lund, Bryan Serrels, Adam Byron, Rhoanne C. McPherson, Alexander von Kriegsheim, Laura Gómez-Cuadrado, Marta Canel, Morwenna Muir, Jennifer E. Ring, Eleni Maniati, Andrew H. Sims, Jonathan A. Pachter, Valerie G. Brunton, Nick Gilbert, Stephen M. Anderton, Robert J.B. Nibbs, and Margaret C. Frame

SUPPLEMENTAL EXPERIMENTAL PROCEDURES

Animal Procedures

All experiments involving animals were carried out in accordance with the UK Coordinating Committee on Cancer Research guidelines by approved protocol (Home Office Project Licence no. 60/4248).

Generation of FAK Nuclear Localization Mutant (FAK-NLS)

Point mutations were introduced into wild-type FAK (FAK-wt) at R177A, R178A, K190A, K191A, K216A, and K218A using site directed mutagenesis. Specifically, 20 ng of pWZL (Hygro) FAK-wt DNA template was mixed with 0.5 μM of each primer in PfuUltra Hotstart master mix (Stratagene) and subjected to PCR with cycle conditions as follows: 95°C (30 s), 18 cycles; 95°C (30 s), 50°C (1 min), 68°C (12 min), final incubation 4°C (infinite). Following PCR, samples were incubated with 10 units of DpnI restriction enzyme at 37°C for 1 hour. The resulting product was transformed into TOP10 chemically competent bacteria (Life Technologies) and plated onto ampicillin agar plates. Colonies were selected and expanded in L broth containing ampicillin overnight, DNA was isolated using a QIAprep DNA miniprep kit (Qiagen), and mutational status was confirmed by sequencing. Three rounds of mutagenesis were required to generate FAK-NLS (R177A, R178A, K190A, K191A, K216A, K218A). Primer sequences were as follows:

FAK R177A/R178A forward,
CTAGGTTGCCTTGAAATCGCGGCATCCTACGGAGAGATGAG;
FAK R177A/R178A reverse,
CTCATCTCTCCGTAGGATGCCGCGATTTCAAGGCAACCTAG;
FAK K190A/K191A forward,
GAGGCAATGCATTAGAGGCGGCATCCAACTATGAAGTGCTAG;
FAK K190A/K191A reverse,
CTAGCACTTCATAGTTGGATGCCGCCTCTAATGCATTGCCTC;
FAK K216A/K218A forward,
GAAGAGTTTGCTAGATTCAGTGGCGGCCGCAACACTACGAAAATTAATC;
FAK K216A/K218A reverse,
GATTAATTTTCGTAGTGTTGCGGCCGCCACTGAATCTAGCAAACTCTTC.

Cell Lines

Isolation and generation of the FAK SCC cell model is described in detail in Serrels et al. (Serrels et al., 2012). Briefly, SCCs were induced in K14*Cre*ER *FAK*$^{flox/flox}$ mice on the FVB background using the DMBA/TPA two-stage skin chemical carcinogenesis protocol and cells

isolated. Following treatment with 4-hydroxytamoxifen, a FAK-null (FAK-/-) cell clone was isolated, and retroviral transduction was used to stably re-express FAK-wt, FAK kinase-dead (FAK-kd), and FAK-NLS. Briefly, Phoenix Ecotropic cells were transfected with pWZL (Hygro) FAK constructs using Lipofectamine 2000 (Life Technologies) according to manufacturer's instructions. Twenty-four hours post-transfection, cell culture supernatant was removed, filtered through a 0.45-μm Millex-HA filter (Millipore), diluted at a 1:1 ratio in normal SCC cell culture medium, supplemented with 5 μg/ml polybrene, and added to SCC FAK-/- cells for 24 hours. A total of two rounds of infection were performed to generate each cell line. Cells were cultured at 37°C in Glasgow minimum essential medium (MEM) (Sigma-Aldrich) supplemented with 2 mM L-glutamine, MEM vitamins, 1 mM sodium pyruvate (all Sigma-Aldrich), MEM amino acids, and 10% fetal bovine serum (FBS) (both Life Technologies), and maintained under selection using 0.25 mg/ml hygromycin.

Western Blotting

To prepare whole cell lysates, cells were washed twice in ice-cold phosphate-buffered saline (PBS) and lysed in RIPA buffer [50 mM Tris-HCl (pH 7.6), 150 mM sodium chloride, 1% Triton X-100, 0.5% sodium deoxycholate, 0.1% SDS, cOmplete Ultra protease inhibitor cocktail (Roche), PhosSTOP phosphatase inhibitor cocktail (Roche)]. Cytoplasmic and nuclear extracts were prepared as described previously (Lim et al., 2008) and summarized below. Lysates were resolved by 4–15% Bis-Tris gel electrophoresis (Biorad), and proteins were transferred to nitrocellulose then blocked [5% bovine serum albumin in PBS–Tween 20 (BSA/PBS-T)] and probed with either anti-Bcl2 (Cell Signaling Technology), anti-FAK (Becton Dickinson), anti-PARP (Cell Signaling Technology), anti-GAPDH (Cell Signaling Technology), rabbit anti-TAF9 (Abcam), or anti-tubulin (Becton Dickinson) primary antibodies (all 1:1000 in 5% BSA/PBS-T). Bound antibody was detected by incubation with anti-rabbit or anti-mouse DyLight800-conjugated secondary antibody or HRP-conjugated secondary antibody (Cell Signaling Technology) and visualized using a Licor Odyssey imaging system or a BioRad ChemiDoc MP Imaging System.

Nuclear Fractionation

SCC FAK-wt and SCC FAK-/- cells were grown on tissue culture dishes for 48 hours at 37°C prior to fractionation. Keratinocyte cultures were prepared from two-day-old FVB pups as described previously (McLean et al., 2004). Keratinocytes pooled from seven pups were grown on two tissue culture dishes for 72 hours at 37°C prior to fractionation. For subcellular fractionation, cells were washed twice with ice-cold PBS, scraped into ice-cold cyto buffer [10 mM Tris-HCl (pH 7.5), 100 mM NaCl, 0.05% NP-40, 3 mM $MgCl_2$, 1 mM EGTA, cOmplete Ultra protease inhibitor cocktail (Roche), PhosSTOP phosphatase inhibitor cocktail

(Roche)] and incubated for 5 min on ice. Cytosolic supernatants were collected following centrifugation at 800 × *g* for 5 min at 4°C. Nuclear pellets were washed twice in cyto buffer, re-suspended in RIPA buffer and incubated for 15 min at 4°C. Nuclear supernatants were collected following centrifugation at 18,000 × *g* for 15 min at 4°C. For whole cell lysates, cells were washed twice with ice-cold PBS, scraped into RIPA buffer, and incubated for 15 min at 4°C. Clarified supernatants were collected by centrifugation at 18,000 × *g* for 15 min at 4°C. Protein concentration was determined by BCA protein assay (Thermo Fisher Scientific), and Western blot analysis performed as described above.

Subcutaneous Tumor Growth

Cells were injected subcutaneously into both flanks of either CD-1 nude mice (0.25×10^6 cells) or FVB mice (1×10^6 cells) and tumor growth measured twice-weekly using calipers. Animals were sacrificed when tumors reached maximum allowed size, or more commonly when signs of ulceration were evident. For studies involving treatment with VS-4718, drug was prepared in 0.5% carboxymethyl cellulose containing 0.1% Tween 80 (Sigma-Aldrich), and mice were treated at 75 mg/kg BID by oral gavage. Animals were visually monitored for signs of toxicity and weighed prior to each dose of VS-4718. No signs of toxicity were observed. Group sizes ranged from 3–5 mice, each bearing two tumors, and tumor volume was calculated in Excel (Microsoft) using the formula $v = 4/3\pi r^3$. Statistics and graphs were calculated using Prism (GraphPad).

Tumor Growth Following Re-challenge

1×10^6 SCC FAK-/- cells were injected subcutaneously into the left flank of FVB mice and tumor growth measured twice-weekly as described above. Following tumor regression, mice were left for 7 days before being challenged with 1×10^6 SCC FAK-wt or FAK-/- cells injected subcutaneously into the right flank. Control groups were injected subcutaneously into both flanks at day 28 using mice that had not been pre-challenged with SCC FAK-/- cells. Tumor volume was calculated as described above.

$CD4^+$, $CD8^+$, and $CD25^+$ T-cell Depletion

Anti-mouse CD4 (GK1.5, ATCC TIB-207) and CD8 (2.43, ATCC TIB-210) depleting antibodies were purified in-house from conditioned supernatant using a Melon Gel IgG purification kit or purchased from eBioscience (anti-CD4 clone GK1.5, anti-CD8 clone 53-6.7). Rat IgG isotype control and anti-mouse CD25 (clone PC61.5) antibodies were purchased from eBioscience. T-cell depletion was achieved following intra-peritoneal (IP) injection of 150 µg of depleting antibody (same for all antibodies) into female age-matched FVB mice for 3 consecutive days, and maintained by further IP injection at 3-day intervals

until the study was terminated. 1 × 10^6 SCC FAK-wt or FAK-/- cells were injected subcutaneously into both flanks 6 days after initial antibody treatment, and tumor growth was measured twice-weekly as described above. The extent of T-cell depletion was determined at the end of the study using FACS analysis of disaggregated spleen and thymus tissue (Figure S1).

FACS Analysis of Immune Cell Populations

Tumors established following injection of 1 × 10^6 SCC cells into both flanks of FVB mice were removed at day 7 into RPMI (Sigma-Aldrich) supplemented with 10% FBS (Life Technologies). Tumor tissue was mashed into a pulp using a scalpel and re-suspended in DMEM (Sigma-Aldrich) supplemented with 2 mg/ml collagenase D (Roche). Samples were incubated for 1 hour at 37°C, pelleted by centrifugation at 1600 rpm for 5 min at 4°C, re-suspended in 5 ml of red blood cell lysis buffer (Pharm Lysis Buffer, Becton Dickinson) for 5 min at 37°C, pelleted by centrifugation at 1600 rpm for 5 min at 4°C, re-suspended in PBS and passed through a 70-µm cell strainer. The resulting single cell suspension was pelleted by centrifugation at 1600 rpm for 5 min at 4°C and re-suspended in FACS buffer (PBS containing 1% FBS and 0.1% sodium azide). This step was repeated a total of three times. A sample of the suspension was counted using trypan blue exclusion and the remaining cell suspension pelleted by centrifugation at 1600 rpm for 5 min at 4°C and re-suspended in FACS buffer at a concentration of 1 × 10^6 viable cells/ml. One hundred microlitres of each sample were pipetted into each well of a 96-well plate and the plate centrifuged at 1600 rpm for 5 min at 4°C. Cell pellets were re-suspended in 50 µl of Fc block [1:200 dilution of Fc antibody (eBioscience) in FACS buffer] and incubated for 15 min at 4°C. Fifty microlitres of antibody mixture [1:200 dilution of antibodies (listed in Table S2) in FACS buffer] were added to each well and the samples incubated for 30 min in the dark at 4°C. The plate was then centrifuged at 1600 rpm for 5 min at 4°C and the cells re-suspended in FACS buffer and analyzed using a BD FACS Aria II. Regulatory T-cell staining was performed using a Treg staining kit (eBioscience). Staining of spleen and thymus tissue was performed as above. Data analysis was performed using FlowJo software. All antibodies were from eBioscience. For absolute counting, the same protocol as above was used, except tumors were weighed following surgical removal, and prior to FACS analysis samples were resuspended in varying volumes of FACS buffer (dependent on weight) containing CountBright Absolute Counting Beads (Life Technologies). Statistics and graphs were calculated using Prism (GraphPad).

Gene Expression Profiling

RNA was prepared from SCC FAK-wt and SCC FAK-/- cells using an RNAeasy kit (Qiagen) according to the manufacturer's instructions. RNA was analysed using the GeneChip Mouse

Genome 430 2.0 Array (Affymetrix). Gene expression data were analyzed using packages within Bioconductor (Gentleman et al., 2004) implemented in the R statistical programming language. The gene expression data were summarised from CEL files using Ensembl gene identifiers and normalized using the Robust Multi-array Average algorithm (Wu and Irizarry, 2004) within the 'affy' package. The data discussed in this publication have been deposited in NCBI's Gene Expression Omnibus (Edgar et al., 2002) and are accessible through GEO Series accession number GSE71662. Data for differentially expressed genes ($p < 0.01$) were median centered and subjected to unsupervised agglomerative hierarchical clustering on the basis of Euclidean distance computed with a complete-linkage matrix using Cluster 3.0 (C Clustering Library, version 1.37) (de Hoon et al., 2004). Clustering results were visualized using Java TreeView (version 1.1.1) (Saldanha, 2004). Functional enrichment analysis against the mouse genome background was performed using ToppGene (Chen et al., 2009).

Quantitative RT^2-PCR Array Analysis of Cytokine, Chemokine, and Chemokine Receptor Expression

RNA prepared from SCC cells was analyzed using the mouse cytokine and chemokine RT^2 Profiler PCR Array (PAMM-150Z; Qiagen) and that from isolated Tregs (isolated using Treg isolation kit, 130-091-041; Miltenyi Biotech) was analyzed using the mouse chemokine and receptor array (PAMM-022Z; Qiagen) according to the manufacturer's instructions. Relative gene expression ($2^{-\Delta Ct}$) values were log transformed, median centered and subjected to hierarchical clustering as for microarray analysis. For interaction network analysis, an interactome of chemokine ligands and receptors was constructed using the IUPHAR/BPS Guide to Pharmacology database (Alexander et al., 2013; Pawson et al., 2014) and curated from the literature (Bachelerie et al., 2014), onto which expression data for detected genes were mapped and visualized using Cytoscape (version 3.0.2) (Shannon et al., 2003). In addition, expression of selected cytokine and chemokine genes was assessed by standard quantitative RT-PCR. TGFβ2-specific primers were purchased from Qiagen (catalog no. PPM02992A). Ccl5 primers used were: forward, CCCTCACCATCATCCTCACT; reverse, CCTTCGAGTGACAAACACGA. Cxcl10 primers used were: forward, CCCACGTGTTGAGATCATTG; reverse, CACTGGGTAAAGGGGAGTGA. B2M primers used were: forward, GGGAAGCCGAACATACTGAA; reverse, TGCTTAACTCTGCAGGCGTAT. Briefly, a reaction mix consisting of 10 µl SensiFAST SYBR Hi-ROX reagent (Bioline), 0.4 µl of 10 µM stock forward primer, 0.4 µl of 10 µM stock reverse primer, 4.4 µl water, and 4 µl of 20 ng/µl stock random hexamer primed cDNA was run on a Rotor-Gene qRT-PCR machine (Qiagen) using the following cycling conditions: 94°C for 10 min; 40 cycles of 94°C for 10 s, 57°C for 20 s, 72°C for 20 s; and 72°C for 7 min.

Analysis was performed using Rotor-Gene software, and expression relative to B2M was calculated using Excel (Microsoft).

shRNA-mediated TGFβ2 and Ccl5 Knockdown

To generate lentiviral particles, 2 × 10^6 HEK293FT cells were transfected with a mixture of 10 µg shRNA (RMM4534-EG21808; GE Healthcare), 6.5 µg HIV, and 3 µg VSVG plasmid DNA using Lipofectamine 2000 as per manufacturer's protocol. Twenty-four hours post-transfection, medium was removed and filtered through a 0.45-µm Millex-AC filter (Millipore) and mixed at a 1:1 ratio with normal SCC growth medium. This mixture was supplemented with polybrene to a final concentration of 5 µg/ml and added to SCC cells for 24 hours. Cells were subject to two rounds of lentiviral infection prior to selection with puromycin at a final concentration of 2 µg/ml. All shRNA constructs used were part of the pLKO lentiviral TRC library (GE Healthcare).

Preparation and Fractionation of Nuclei and Chromatin

Nuclei were prepared as previously described (Gilbert et al., 2003) but with a reduced concentration (0.05%) of NP-40 in nuclei buffer B. Soluble chromatin was prepared as described previously (Gilbert et al., 2004) and fractionated on a 5-ml sucrose step gradient to separate soluble and chromatin-associated nuclear proteins. Essentially, the soluble chromatin was layered over 10% and 50% sucrose in TEP80 buffer [10 mM Tris-HCl (pH 8.0), 1 mM EDTA, 80 mM NaCl, 100 µM PMSF] and centrifuged at 50,000 rpm for 1 h 50 min in an MLS-50 rotor (Beckman). Five hundred–microlitre fractions were collected by upward displacement in a gradient fractionator (Teledyne ISCO). DNA was recovered from fractions using a PCR purification kit (Qiagen) and subjected to agarose (1.5%) gel electrophoresis. Protein was purified using TCA precipitation. Specifically, one volume of TCA stock solution was mixed with four volumes of protein solution, incubated at 4°C for 10 min, and spun at 14,000 rpm for 5 min at 4°C. The supernatant was discarded, and the pellet was washed twice with ice-cold acetone and dried for 5 min at room temperature. Samples were analysed by SDS-PAGE and western blotted using anti-FAK (Cell Signalling Technologies), anti-HP1α (Millipore), and anti-histone H3 (Cell Signalling Technologies) antibodies.

Proteomic Analysis of Nuclear FAK Protein Complexes

Two milligrams of nuclear lysates were incubated with 10 µl agarose-conjugated anti-FAK antibody (clone 4.47; Merck Millipore) overnight at 4°C with rotation. Beads were washed twice with ice-cold RIPA buffer and twice with ice-cold PBS. Bead-bound complexes from biological triplicate experiments were subjected to on-bead proteolytic digestion, desalting

and liquid chromatography–tandem mass spectrometry in technical duplicate as described previously (Turriziani et al., 2014). Mean label-free MS intensities were calculated from technical duplicate data acquisitions for each biological replicate. Peptide and protein false discovery rates were set to 1%. Proteins enriched from SCC FAK-wt nuclei by at least two-fold over SCC FAK-/- control nuclei ($p < 0.05$) were considered specifically isolated by FAK immunoprecipitation. For interaction network analysis, Ccl5 transcription factors were extracted from the DECODE database (Qiagen) (most relevant transcription factors predicted to bind between 20 kb upstream and 10 kb downstream of the Ccl5 transcription starting site for human, mouse and rat) and used to seed a network of 1000 transcription factor–related proteins using the GeneMANIA plugin (version 2014-08-12-core; human interactions) in Cytoscape. Proteins specifically isolated in nuclear FAK protein complexes were mapped onto the interactome, and those with physical or predicted direct or indirect interactions with Ccl5 transcription factors were analyzed using the NetworkAnalyzer plugin (version 2.7) in Cytoscape.

CD8 T-cell Fluorescent Immunohistochemistry

SCC tumors were surgically removed 7 days post-implantation and snap-frozen in a cryovial by submersing in liquid nitrogen. Tumor sections were cut using a cryostat and stored at –80°C. Prior to staining, sections were moved to –20°C overnight. Slides were air-dried for 30 min, fixed with ice-cold acetone (Sigma-Aldrich) for 10 min at room temperature, and washed three times for 5 min each in PBS. Tumor sections were outlined using a hydrophobic barrier pen (DAKO), then incubated with mouse background block for 1 h at room temperature. Samples were incubated with primary antibody [anti-mouse CD8α (clone 53-6.7), rat IgG2a; R&D Systems; 1:100 dilution in 5% goat serum–2.5% BSA in PBS] for 1 h at room temperature, then washed three times with PBS-T, each for a period of 5 min, followed by three 5-min PBS washes. Samples were incubated with secondary antibody [Alexa Fluor 647 goat anti-rat IgG (H+L) (Life Technologies), 1:200 dilution] for 1 h at room temperature in the dark, then subjected to three 5-min washes with PBS-T, followed by one 5-min wash with PBS and one wash with sterile water. Samples were mounted under a coverslip (Thermo Scientific) using ProLong Gold Antifade Mount containing DAPI (Life Technologies) and imaged using an Olympus FV1000 confocal microscope. Images were acquired using an UPLSAPO 20× lens. DAPI and Alexa Fluor 647 were imaged using 405-nm and 635-nm excitation lasers. Three-dimensional image stitching was performed using the Olympus multi-site time-lapse module, with the final image comprising an 8 × 8 matrix with 20% overlap between adjacent images. Images were rendered using Imaris (Bitplane).

SUPPLEMENTAL TABLES

Table S2. Markers and Antibody Combinations Used to Define Immune Cell Populations, Related to Experimental Procedures

(A) T-cell subsets for FACS analysis. (B) Macrophage subsets for FACS analysis. (C) MDSC subsets for FACS analysis. (D) Treg subsets for FACS analysis.

A

Population	Markers
$CD4^+$ T cell	$CD45^+$ $CD3^+$ $CD4^+$ $CD8^-$
$CD4^+$ Central Memory T cell	$CD45^+$ $CD3^+$ $CD4^+$ $CD8^-$ $CD44^{hi}$ $CD62L^{hi}$
$CD4^+$ Effector T cell	$CD45^+$ $CD3^+$ $CD4^+$ $CD8^-$ $CD44^{hi}$ $CD62L^{low}$
$CD8^+$ T cell	$CD45^+$ $CD3^+$ $CD4^-$ $CD8^+$
$CD8^+$ Central Memory T cell	$CD45^+$ $CD3^+$ $CD4^-$ $CD8^+$ $CD44^{hi}$ $CD62L^{hi}$
$CD8^+$ Effector T cell	$CD45^+$ $CD3^+$ $CD4^-$ $CD8^+$ $CD44^{hi}$ $CD62L^{low}$

Marker	Fluorophore	Filter Settings
Viability	Viability 506	405_525/50
CD45	e450	405_450/50
CD3	FITC	488_525/50
CD8	PE	561_582/15
CD4	e647	640_617/14
CD62L	PE-Cy7	561_780/60
CD44	PerCP - Cy5.5	488_685/35

B

Population	Markers
Inflammatory Macrophage (iMac)	$CD45^+$ $CD11b^+$ $F4/80^+$ $Ly6C^+$
Tissue-Resident Macrophage (Mac)	$CD45^+$ $CD11b^+$ $F4/80^+$ $Ly6C^-$

Marker	Fluorophore	Filter Settings
Viability	Viability 506	405_525/50
CD45	e780	640_780/60
F4/80	FITC	488_525/50
CD11b	PerCP - Cy5.5	488_685/35
Ly6C	e450	405_450/50

C

Population	Markers
M-MDSC	$CD45^+$ $CD11b^+$ $F4/80^-$ $Ly6C^{hi}$ $Gr1^{lo}$
G-MDSC	$CD45^+$ $CD11b^+$ $F4/80^-$ $Ly6C^{int}$ $Gr1^{hi}$

Marker	Fluorophore	Filter Settings
Viability	Viability 506	405_525/50
CD45	e780	640_780/60
CD11b	PerCP - Cy5.5	488_685/35
F4/80	PE-Cy7	561_780/60
Ly6C	e450	405_450/50
Gr1	FITC	405_450/50

D

Population	Markers
Treg	$CD4^+$ $FoxP3^{hi}$ $CD25^{hi}$

Marker	Fluorophore	Filter Settings
CD4	PerCP - Cy5.5	488_685/35
CD25	PE	561-582_15
FoxP3	FITC	405_450/50

SUPPLEMENTAL REFERENCES

Alexander, S.P., Benson, H.E., Faccenda, E., Pawson, A.J., Sharman, J.L., McGrath, J.C., Catterall, W.A., Spedding, M., Peters, J.A., Harmar, A.J.; CGTP Collaborators, et al. (2013). The Concise Guide to PHARMACOLOGY 2013/14: overview. Br. J. Pharmacol. *170*, 1449–1458.

Bachelerie, F., Graham, G.J., Locati, M., Mantovani, A., Murphy, P.M., Nibbs, R., Rot, A., Sozzani, S., and Thelen, M. (2014). New nomenclature for atypical chemokine receptors. Nat. Immunol. *15*, 207–208.

Chen, J., Bardes, E.E., Aronow, B.J., and Jegga, A.G. (2009). ToppGene Suite for gene list enrichment analysis and candidate gene prioritization. Nucleic Acids Res. *37*, W305–W311.

de Hoon, M.J., Imoto, S., Nolan, J., and Miyano, S. (2004). Open source clustering software. Bioinformatics *20*, 1453–1454.

Edgar, R., Domrachev, M., and Lash, A.E. (2002). Gene Expression Omnibus: NCBI gene expression and hybridization array data repository. Nucleic Acids Res. *30*, 207–210.

Gentleman, R.C., Carey, V.J., Bates, D.M., Bolstad, B., Dettling, M., Dudoit, S., Ellis, B., Gautier, L., Ge, Y., Gentry, J., Hornik, K., Hothorn, T., Huber, W., Iacus, S., Irizarry, R., Leisch, F., Li, C., Maechler, M., Rossini, A.J., Sawitzki, G., Smith, C., Smyth, G., Tierney, L., Yang, J.Y., and Zhang, J. (2004). Bioconductor: open software development for computational biology and bioinformatics. Genome Biol. *5*, R80.

Pawson, A.J., Sharman, J.L., Benson, H.E., Faccenda, E., Alexander, S.P., Buneman, O.P., Davenport, A.P., McGrath, J.C., Peters, J.A., Southan, C., Spedding, M., Yu, W., Harmar, A.J.; NC-IUPHAR. (2014). The IUPHAR/BPS Guide to PHARMACOLOGY: an expert-driven knowledgebase of drug targets and their ligands. Nucleic Acids Res. *42*, D1098–D1106.

Saldanha, A.J. (2004). Java Treeview--extensible visualization of microarray data. Bioinformatics *20*, 3246–3248.

Shannon, P., Markiel, A., Ozier, O., Baliga, N.S., Wang, J.T., Ramage, D., Amin, N., Schwikowski, B., and Ideker, T. (2003). Cytoscape: a software environment for integrated models of biomolecular interaction networks. Genome Res. *13*, 2498–2504.

Wu, Z. and Irizarry, R.A. (2004). Preprocessing of oligonucleotide array data. Nat. Biotechnol. *22*, 656–658; author reply 658.
